# Development and Phantom Validation of a Small-Form-Factor SWIR Emitter Probe for Hydration-Sensitive Spatial-Ratio Measurements in Gelatin–Intralipid Phantoms

**DOI:** 10.3390/s26072020

**Published:** 2026-03-24

**Authors:** Georgei Farouq, Devang Vyas, Amir Tofghi Zavareh

**Affiliations:** 1 Department of Engineering Technology and Industrial Distribution, Texas A&M University, College Station, TX 77843, USA; georgeifarouq@tamu.edu; 2 Department of Biomedical Engineering, Texas A&M University, College Station, TX 77843, USA; devvyas83@tamu.edu; 3 Center for Remote Health Technologies and Systems, Texas A&M University, College Station, TX 77843, USA

**Keywords:** short-wave infrared spectroscopy (SWIRS), hydration sensing, gelatin–Intralipid phantom, diffuse reflectance, spatially resolved spectroscopy, Monte Carlo lookup tables, small-form-factor sensor

## Abstract

Non-invasive assessment of tissue water content is clinically relevant for edema detection, fluid management, and monitoring of local inflammation. In the short-wave infrared (SWIR), water exhibits strong absorption near 1450 nm with a secondary band near 1650 nm, enabling hydration-sensitive reflectance measurements. However, many SWIR systems rely on spectrometers or high-power broadband sources, limiting translation to compact or wearable platforms. We present a compact SWIR diffuse-reflectance probe built from small-form-factor components using four discrete LEDs (1450 nm and 1650 nm) and a single photodetector to acquire spatially resolved measurements at two source–detector separations (4.5 mm and 7 mm). Probe-geometry-matched Monte Carlo simulations were used to generate lookup tables relating reduced scattering to same-wavelength spatial ratios. A diffusion-based forward model was then used to perform a calibration-anchored water-fraction consistency analysis. Eight gelatin–Intralipid phantoms spanning two scattering conditions and formulation-defined water fractions were evaluated. Spatial-ratio signatures were repeatable and monotonic with nominal water fraction, yielding a mean absolute percent error of 1.55% and a maximum absolute percent error of 3.33% under absorption-consistent conditions. These results demonstrate the feasibility of compact SWIR ratio sensing for controlled hydration changes in tissue-mimicking phantoms and provide a modeling framework for future extension to unknown or in vivo samples.

## 1. Introduction

Non-invasive assessment of tissue water content is clinically relevant for edema evaluation, fluid-management guidance, and monitoring of local inflammation [1]. Conventional bedside assessments (e.g., pitting and skin turgor) are subjective and operator dependent [2,3]. Optical methods in the near-infrared (NIR) and short-wave infrared (SWIR) are attractive because tissue attenuation in these bands is strongly influenced by water absorption while remaining compatible with small-form-factor optoelectronics [4,5].

In the SWIR, water exhibits a strong O–H combination band near 1450 nm and a weaker band near 1650 nm [5,6,7]. Above approximately 1300 nm, absorption from dominant visible/NIR chromophores (e.g., hemoglobin and melanin) is reduced, and water can become a principal contributor to attenuation in soft tissue [5,8,9,10]. Diffuse reflectance at water-sensitive wavelengths therefore decreases with increasing bulk water content [2,9]. The present work uses 1450 nm for high hydration sensitivity and 1650 nm as a secondary band with lower absorption, providing a practical trade-off between sensitivity and sampling depth [4,8,9,10]. More strongly absorptive bands near 1950 nm offer limited penetration and impose substantial implementation constraints for compact reflectance sensing [4,5,8,10,11].

Many reported hydration-related NIR/SWIR systems rely on spectrometers, tunable sources, or multispectral imaging architectures that increase size, cost, and power consumption [2,4,8,9,12,13]. Recent studies in Fluorescence Molecular Tomography (FMT) have utilized the NIR-II/SWIR window to achieve deeper 3D tomographic reconstruction and higher resolution by minimizing background autofluorescence [14]. Furthermore, advancements in Spatial Frequency Domain Imaging (SFDI) have demonstrated that SWIR wavelengths provide a melanin-agnostic platform for quantifying biomarkers such as water and lipid concentrations in vivo [15]. Discrete-wavelength reflectance sensing provides an alternative path to miniaturization by using compact emitters at selected water bands and a single photodetector [6,16,17,18].

To reduce sensitivity to subject- and coupling-dependent variability (e.g., scattering changes due to microstructure, contact pressure, and boundary effects), spatially resolved measurements at multiple source–detector separations (SDSs) can be used [19,20,21]. Because absorption and reduced scattering affect the radial decay of diffuse reflectance differently, multi-SDS ratios provide additional constraints for interpreting hydration-sensitive changes compared with a single-distance measurement [19,20,21,22,23].

**Figure 1:**
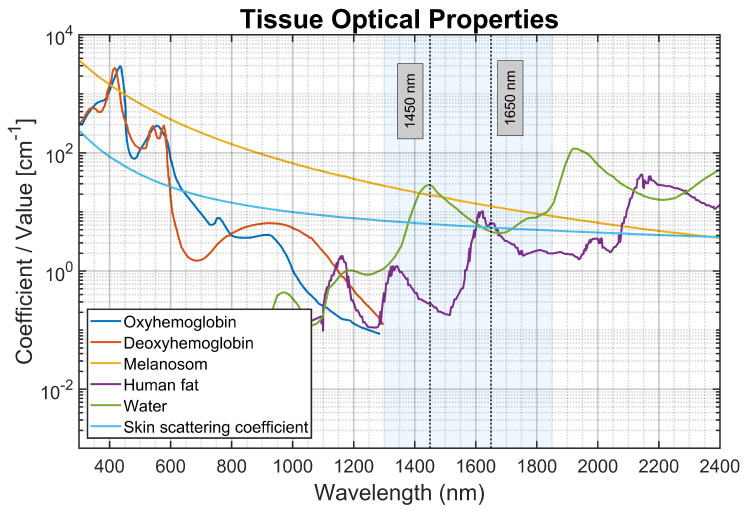
Absorption and scattering properties of major biological tissue chromophores from the visible to short−wave infrared (SWIR) spectrum. Data was captured from [29].

Here, we develop and validate a compact SWIR reflectance probe that acquires two-wavelength, two-SDS measurements (1450/1650 nm; 4.5/7 mm) in controlled gelatin–Intralipid phantoms spanning recipe-defined water fractions and multiple scattering conditions [24,25,26,27,28]. To visually support the choice of 1450 nm and 1650 nm, Figure 1 shows the absorption coefficients ($\mu_{a}$) of the main tissue chromophores—water, melanosome, lipids, oxy-hemoglobin, and deoxy-hemoglobin—from 300 to 2400 nm, as well as the skin scattering coefficient ($\mu_{s}$). Operating above 1300 nm effectively reduces interference from melanin and hemoglobin, the main absorbers in the visible and near-infrared (NIR) ranges. The 1450 nm band specifically targets the strong O-H combination peak of water, which is highly sensitive to hydration. In contrast, the 1650 nm wavelength has lower water absorption, allowing a greater contribution from longer photon paths to the far separation. This provides a crucial limit to differentiate between absorption-driven and scattering-driven signal decay [29].

Monte Carlo simulations matched to the probe geometry are used to generate lookup tables for interpreting spatial ratios [30,31,32]. In this prototype, detection is performed with an amplified laboratory InGaAs photodetector module [4,6,16].

The objective of this study is to validate that a compact SWIR probe using small-form-factor emitters produces repeatable, monotonic spatial-ratio signatures of hydration in controlled gelatin–Intralipid phantoms with recipe-defined water fractions [2,6,9,16,26]. Specifically, we evaluate (i) the monotonic dependence of same-wavelength spatial ratios on nominal phantom water fraction under fixed acquisition conditions, and (ii) the agreement of an absorption-assigned, probe-matched inversion pipeline with the recipe-defined values as a consistency check, rather than blind estimation of $\mu_{a}\left(\lambda \right)$ or $f_{w}$ from reflectance alone [19,21,32]. Accordingly, the results presented herein are intended to demonstrate internal consistency and methodological stability under controlled phantom conditions rather than provide a framework for blind recovery of unknown samples.

This workflow is critical as it decouples instrument response from model-based inference, rigorously benchmarks hydration sensitivity against a ground-truth optical standard, and establishes a physically constrained calibration pathway that strengthens confidence in subsequent translation to heterogeneous biological tissue where $\mu_{a,\lambda }$ and scattering cannot be independently specified.

## 2. Materials and Methods

Section 2 describe: (i) gelatin–Intralipid phantom fabrication with recipe-defined (nominal) $f_{w,\mathrm{true}}$, (ii) probe-geometry-matched MC LUT generation used to invert $\mu_{s}^{\prime }$ from spatial ratios conditional on assigned $\mu_{a}\left(\lambda \right)$, (iii) the benchtop measurement hardware and optical interface, and (iv) a ratio-based inversion used as a calibration-anchored consistency check rather than blind recovery for unknown samples [20,21,32].

### 2.1. Phantom Production Pipeline and Rationale

#### 2.1.1. Motivation for In Vitro Phantom Use

Gelatin-based phantoms provide a controllable, repeatable test medium for early-stage probe validation prior to in vivo studies, where inter-subject variability (thickness, hydration, lipids, coupling, and scattering) complicates calibration and characterization [24,33,34]. In this work, phantoms were selected to enable systematic variation of bulk water fraction and scattering while maintaining a stable, contact-compatible surface [24,26].

#### 2.1.2. Gelatin Hydrogel Matrix Rationale

Gelatin hydrogels are widely used tissue-mimicking phantoms because they form mechanically stable, water-rich matrices suitable for repeatable contact measurements [24,26,27,33]. Here, gelatin is treated primarily as a mechanical scaffold; water is the dominant SWIR absorber over 1450–1650 nm [5,9,10].

#### 2.1.3. Use of Intralipid as a Scattering Agent

To emulate tissue-like multiple scattering, Intralipid is used as a standard lipid-emulsion scattering agent with well-characterized optical behavior [25,27,28,35]. Because Intralipid^®^ is an emulsion of lipid droplets in a water background, its absorption is weak and, in practice, close to that of water; therefore, it is treated primarily as a scattering agent in the SWIR bands used here [36]. Phantoms were fabricated with 10% and 20% (*w*/*w*) Intralipid^®^ 20% stock in the final mixture [25,28,35]. Unless otherwise stated, “10% Intralipid” denotes 10% *w*/*w* Intralipid^®^ 20% stock loading in the final mixture (e.g., 1.50 g stock in a 15.0 g batch), and “20% Intralipid” denotes 20% *w*/*w* stock loading (e.g., 3.00 g stock in a 15.0 g batch) [25,35]. The resulting $\mu_{s}^{\prime }$ values, which range from ∼1.48 to $4.50\;{\mathrm{cm}}^{-1}$, were chosen to match the established values for human skin scattering in the SWIR region. This typically falls between 5 and $10\;{\mathrm{cm}}^{-1}$ over the range of 1450 to 1650 nm, as seen in Figure 1. These concentrations help ensure that light transport stays in the strongly diffuse regime at the millimeter-scale separations used here, which are between 4.5 and 7 mm. This condition provides a clear and consistent area for the Monte Carlo Lookup Table (MC-LUT) inversion and the diffusion-based dipole model.

#### 2.1.4. Gelatin Phantom Fabrication

Gelatin phantoms were fabricated by modifying the protocol of Jonasson et al. [26] with gravimetric mass preparation [24,33,34]. Two water-fraction quantities are distinguished: (i) the added-water fraction (added distilled water divided by total batch mass), and (ii) the true water fraction, which also accounts for solvent water in the Intralipid^®^ 20% stock (assumed 80% water by mass) [25,35]. The recipe-defined true water fraction is

(1) $$f_{w,\mathrm{true}}=\frac{m_{\mathrm{water}}+0.8\;m_{\mathrm{IL}}}{m_{\mathrm{total}}},$$

where *m* denotes the mass of water intralipid and total gel, respectively. Gelatin’s intrinsic absorption has been reported to closely track an aqueous background in the near-infrared; accordingly, at the strong SWIR water bands used here (1450 nm and 1650 nm), we neglect gelatin’s intrinsic contribution relative to water and model $\mu_{a}\left(\lambda \right)$ as water-dominant [37]. Throughout, $f_{w,\mathrm{true}}$ is treated as a nominal reference value (dominant uncertainties include Intralipid stock water-content assumption, handling/evaporation during heating, scale repeatability, and any non-homogeneity) [24,27,28].

Each phantom was prepared at a total batch mass of 15.0 g and cast to yield a flat, contact-stable surface [24,33]. Gelatin phantoms were made by first letting gelatin powder spread evenly over the surface of distilled water for 5 min to avoid clumping. Then, the mixture was heated on a hot plate at 55 ^∘^C while stirring with a magnetic stirrer. To achieve complete blending, a stepped stirring method was used: 5 min at 200 RPM, then 5 min increments at 400 RPM and 500 RPM as the solution became liquid, and finishing with a final 5 min stir at 600 RPM. Before adding the Intralipid scattering agent, the temperature was lowered to 45 ^∘^C to prevent thermal stress on the emulsion. After including Intralipid and stirring for another 5 min, the mixture was vacuum-degassed at 27inHg for about 1 min or until all visible air bubbles were eliminated. Finally, the solution was poured into 3D-printed molds and placed in the refrigerator overnight to ensure it fully solidified before measurement [24,27]. The solution was cooled to reduce emulsion stress, and Intralipid^®^ 20% stock was added and mixed. The mixture was vacuum-degassed, then poured into a mold and refrigerated overnight [24,34].

After fabrication, phantoms were sealed to minimize dehydration prior to measurement and were used only if visually homogeneous (no macroscopic creaming/ stratification) [26,27,28]. Phantoms exhibiting visible phase separation were excluded from quantitative analysis because they violate the homogeneous-medium assumption used in both LUT generation and diffusion modeling [24,33]. Representative composition ranges and preparation photos are provided in the Supplementary Materials (Tables S1–S3 and Figure S1); the specific formulations used in this study are listed in Table 1.

### 2.2. Monte Carlo Simulations for Probe-Specific LUT Generation

Monte Carlo (MC) photon-transport simulations (MCmatlab) were used to generate probe-geometry-specific lookup tables (LUTs) that map the reduced scattering coefficient, $\mu_{s}^{\prime }$, to expected diffuse-reflectance observables for the instrument’s two source–detector separations (SDSs) and collection constraints [30,31]. The purpose of the LUTs is to provide a probe-matched reference for how channel magnitudes and, critically, same-wavelength spatial ratios vary with $\mu_{s}^{\prime }$ under specified absorption at each wavelength [21,23,31,32]. Key MC configuration parameters are summarized in the Supplementary Materials (Table S6).

#### 2.2.1. Absorption Parameterization and Scope

Over 1450–1650 nm, absorption in gelatin–Intralipid hydrogels was modeled as water dominated (gelatin and lipid treated as secondary within a simplified model) [5,9,10,33,36,37]. Absorption was assigned as

(2) $$\mu_{a}\left(\lambda \right)\approx f_{w}\;\mu_{a,\mathrm{water}}\left(\lambda \right),$$

where $\mu_{a,\mathrm{water}}\left(\lambda \right)$ was taken from the liquid-water dataset of Hale and Querry [5,7,8] and expressed in ${\mathrm{cm}}^{-1}$. Within each wavelength sweep, $\mu_{a}\left(\lambda \right)$ was held fixed while $\mu_{s}^{\prime }$ was varied; thus the LUT mapping ratio $\to \mu_{s}^{\prime }$ is conditional on the assumed $\mu_{a}\left(\lambda \right)$ [20,21,32]. In this phantom validation study, the absorption coefficient, $\mu_{a}\left(\lambda \right)$, was prescribed based on the recipe-defined (nominal) water fraction, $f_{w,true}$, for each phantom formulation, thereby enabling evaluation of the inversion pipeline under absorption-consistent conditions. The present work is intentionally confined to controlled, optically defined phantoms to establish internal consistency and methodological validity. Extension of this framework to unknown or heterogeneous samples will necessitate a coupled absorption–scattering inversion strategy, in which $\mu_{a}\left(\lambda \right)$ and reduced scattering are jointly estimated from measured reflectance data [32].

#### 2.2.2. Simulation Geometry and Optical Properties

The phantom was modeled as a homogeneous rectangular slab with dimensions 3.5cm×3.5cm×1.2cm, discretized on a 101×101×150 voxel grid to match fabricated gel dimensions [30]. To emulate the experimental optical head and suppress stray-photon collection, a thin optically black “super-absorbing” mask was placed directly above the gel surface, spanning the lateral extent of the domain, with a centered circular through-aperture of diameter 4mm defining the collection aperture [20,32]. Photons intersecting the mask outside the aperture were terminated; photons passing through the aperture were allowed to propagate and be detected, so that the effective field-of-view and collection area were aperture limited, matching the experimental configuration.

The phantom was assigned wavelength-dependent $\mu_{a}\left(\lambda \right)$ and scattering $\mu_{s}\left(\lambda \right)$ with fixed anisotropy g=0.9 [5]. To ensure that $\mu_{s}^{\prime }$ is the controlled sweep variable,

(3) $$\mu_{s}=\frac{\mu_{s}^{\prime }}{1-g}.$$

A water-dominant refractive index (n≈1.33) was used as a first-order approximation for boundary behavior in the homogeneous medium model; sensitivity to boundary assumptions is addressed in the Discussion [22,38].

#### 2.2.3. Finite Source and Aperture-Limited Collection (Probe Emulation)

Illumination was modeled as a finite-area LED source [16]. For each wavelength channel and separation $\rho \in \{\rho_{c},\rho_{f}\}$, the LED emission center was positioned at lateral offset ρ relative to the detector/aperture axis on the sample surface, with launch positions sampled uniformly over a finite emitting area consistent with the LED package dimensions [16,20]. Launch directions followed a datasheet-matched, generalized Lambertian divergence.

Photon collection was implemented using the MCmatlab light-collector model co-axial with the aperture at the surface [30,32]. To emulate aperture/tunnel throughput, the collector active diameter was set to 4mm and photons were additionally filtered by an acceptance cone consistent with the cylindrical tunnel geometry [19,20,32].

#### 2.2.4. LUT Generation, Photon Statistics, and Conditioning

For each phantom condition (10% Intralipid, 20% Intralipid), $\mu_{a}\left(\lambda \right)$ was assigned from the recipe-defined $f_{w,\mathrm{true}}$ and held constant while $\mu_{s}^{\prime }$ was swept over $\mu_{s}^{\prime }\in \left[0.05,\;30\right]\;{\mathrm{cm}}^{-1}$ at each wavelength (1450 nm, 1650 nm) and SDS (4.5 mm, 7 mm) [5,10,25,35]. For each $\left(\lambda ,\rho ,\mu_{s}^{\prime }\right)$ condition, the primary LUT output was the detected photon fraction $p_{\det}$ (detected counts normalized by launched photons) within the probe-emulated collector acceptance [30,31]. Unless otherwise stated, $N=2\times 10^{9}$ photons were launched per condition [30,31].

To avoid ill-conditioned LUT regions dominated by photon starvation (especially at the far SDS in highly absorbing regimes), LUT points with insufficient detected counts were flagged and excluded from inversion and/or rerun at higher *N*, such that the ratio curves used for interpolation remained smooth and monotonic over the inversion-relevant range [32]. Experimentally measured spatial ratios were then mapped to the corresponding MC-derived ratio curves to estimate $\mu_{s}^{\prime }$ consistent with the probe geometry and assigned $\mu_{a}\left(\lambda \right)$; these $\mu_{s}^{\prime }\left(\lambda \right)$ values are used as inputs to the downstream water-fraction consistency-check model [20,32]. Additional statistical details are provided in the Supplementary Materials [30,31].

### 2.3. Hardware Architecture and Circuit Implementation

**Figure 2:**
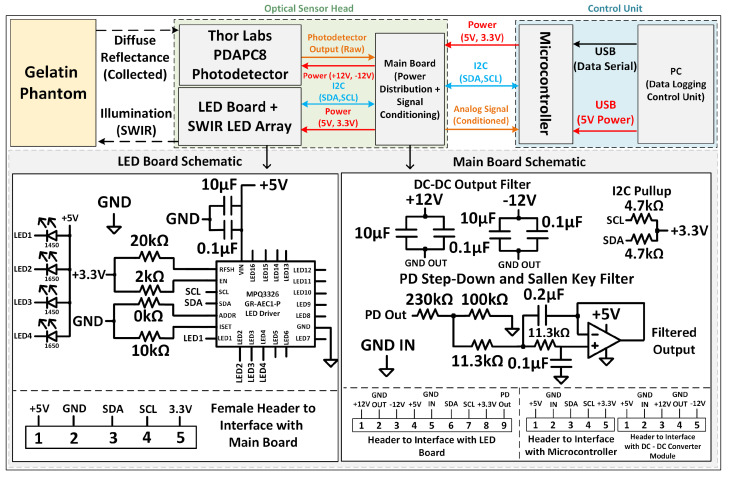
Device block diagram and key schematics. Top: module partitioning and signal/power flow. Bottom: implemented LED–board constant–current drive and main-board power distribution and analog conditioning used to interface the amplified photodetector output to the microcontroller ADC. Red lines denote power connections, blue lines denote I^2^C signals, and orange lines denote analog signals.

The instrument is a four-channel SWIR diffuse-reflectance sensor designed to acquire hydration-sensitive ratio measurements using two wavelengths (1450 nm and 1650 nm) at two source–detector separations (SDSs): a close channel ($\rho_{c}\approx 4.5$ mm) and a far channel ($\rho_{f}\approx 7$ mm). Illumination is time-multiplexed such that only one LED is active at a time. The amplified photodetector output is conditioned on the main board, digitized by a microcontroller ADC, and streamed to a PC over USB for logging and control. Figure 2 summarizes the system architecture and key circuit blocks. Benchtop validation used an amplified laboratory InGaAs photodetector module (Thorlabs PDAPC8).

#### 2.3.1. Module Partitioning and Optical Channels

The system comprises (i) an optical sensor head with four LEDs arranged as two wavelengths at two SDS around a central collection aperture, (ii) a main board providing power distribution and analog conditioning, and (iii) a control unit (microcontroller + PC interface). Mechanical light-control features (aperture/tunnel and baffle geometry) suppress direct-path crosstalk and preferentially collect multiply scattered photons.

Four measurement channels are defined by wavelength and SDS: 1450 nm (close), 1450 nm (far), 1650 nm (close), and 1650 nm (far). The LED drive is provided by a four-channel constant-current sink driver (MPQ3326) controlled via I^2^C (Figure 2). The implemented channel mapping is CH1 = 1450 nm (close), CH2 = 1650 nm (far), CH3 = 1450 nm (far), and CH4 = 1650 nm (close), corresponding to the physical LED placement. A channel-mapping summary is also provided in the Supplementary Materials (Table S4).

#### 2.3.2. Power Distribution and Analog Interface

The main board distributes +5 V and +3.3 V and generates ±12 V rails for the PDAPC8 via a plug-in DC–DC converter (Figure 2). The PDAPC8 analog output is scaled to the ADC input range using a resistive divider and then low-pass filtered prior to digitization. The divider and filter are selected such that the conditioned node remains below ADC saturation while suppressing high-frequency noise for quasi-static intensity measurements under time-multiplexed illumination (the full component values and filter design calculation are provided in the Supplementary Materials). All modules share a common ground reference, and local decoupling is used to reduce coupling of switched LED currents into the analog sensing path.

#### 2.3.3. Time-Multiplexed Measurement Sequence and Acquisition Settings

Measurements are dark-subtracted and normalized to a diffuse reflectance reference (PTFE) to reduce fixed multiplicative differences due to LED radiant power, channel responsivity, and geometry; residual mismatch between reference and sample is treated as a potential contributor to systematic bias. For each gel phantom, one four-channel measurement set (two wavelengths × two SDS) is acquired under consistent probe contact, and a PTFE reference is acquired in close temporal proximity under the same contact geometry. Timing/averaging parameters are summarized in the Supplementary Materials (Table S5).

### 2.4. Mechanical Implementation and Optical Interface

**Figure 3:**
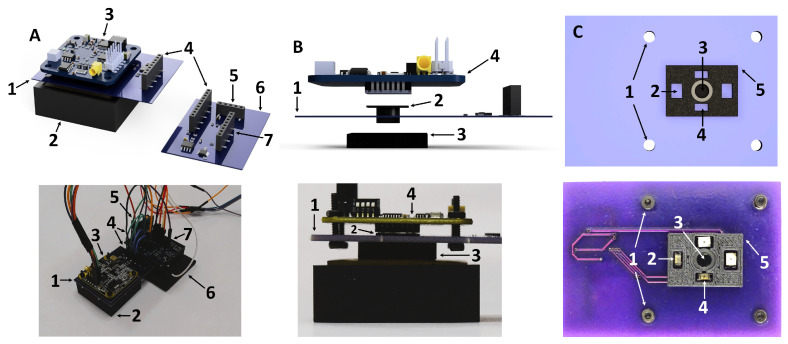
Optical sensor head overview. Top row: CAD renderings showing (**A**) compact sensor head positioned on the gel phantom, (**B**) side view of the assembled optical stack, and (**C**) sample-facing view of the LED emission apertures and central detector tunnel that constrains the photodetector field of view. Bottom row: corresponding photographs. Callouts: (A.1) LED board; (A.2) 3D-printed mold holding the gelatin phantom; (A.3) PDAPC8 photodetector module; (A.4) LED-boar–to–main-board interface; (A.5) female header for the DC–DC module; (A.6) main board; (A.7) female header for interfacing with the microcontroller. (B.1) LED board; (B.2) aperture; (B.3) baffle; (B.4) PDAPC8 photodetector module. (C.1) M2.5 screw apertures securing the PDAPC8 to the LED board; (C.2) aperture for the far LEDs; (C.3) photodetector aperture (PDAPC8); (C.4) aperture for the close LEDs; (C.5) rectangular baffle. The PDAPC8 CAD model was obtained from Thorlabs.

The optical sensor head is a mechanically registered, light-shielded assembly that enforces fixed source–detector geometry while suppressing direct LED–detector crosstalk. Figure 3 summarizes the head stack-up, sample-facing geometry, and device-level assembly context. A Thorlabs PDAPC8 amplified InGaAs photodetector module is clamped to the LED board, providing repeatable alignment between the detector collection axis and the LED source geometry and minimizing optical head height.

#### Detector Aperture/Tunnel for Field-of-View Control

A black 3D-printed aperture/tunnel is sandwiched between the PDAPC8 underside and the LED-board center opening to define aperture-limited collection. The flange contains a 4.0 mm diameter circular opening that defines the effective collection aperture, followed by a cylindrical tunnel extending toward the sample. This geometry limits detector field of view and reduces sensitivity to off-axis/stray light, biasing collection toward multiply scattered (diffuse) photons emerging from the sample. A second black 3D-printed baffle block surrounds the source–detector region on the sample side to suppress shortcut optical paths. The baffle includes a central aperture for the detector tunnel and four LED openings positioned at the nominal SDS used here to reduce line-of-sight or near-surface crosstalk that can confound spatially resolved diffuse-reflectance measurements. Together, the clamped detector mounting, aperture/tunnel, and baffle block provide a repeatable optical interface for spatially resolved measurements. Absolute magnitudes can still vary with contact pressure and coupling; accordingly, downstream analysis emphasizes same-wavelength spatial ratios with same-session reference normalization to reduce sensitivity to multiplicative coupling factors.

### 2.5. Mathematical Inversion and Water-Fraction Consistency-Check Estimation

This section describes the pipeline that converts four electrical measurements (two wavelengths × two SDS) into (i) wavelength-specific reduced scattering estimates and (ii) a calibration-anchored water-fraction consistency-check estimate.

**Figure 4:**
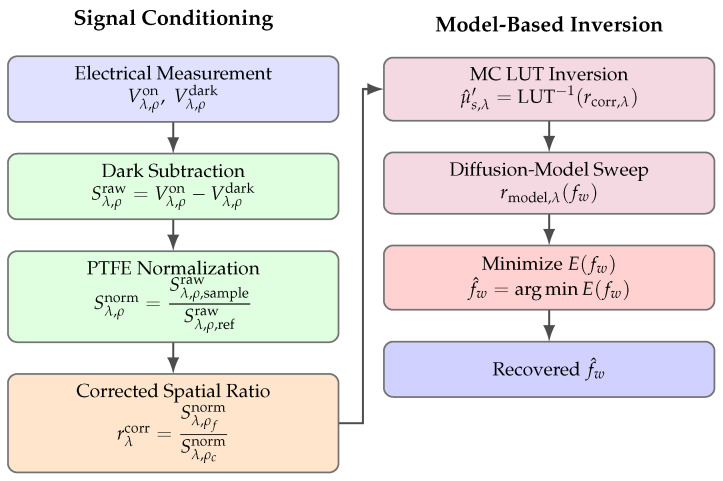
Two-column overview of the inversion pipeline. Left: signal conditioning and formation of corrected spatial ratios. Right: model-based inversion and water-fraction estimation.

The overall inversion workflow is summarized in Figure 4. A probe-matched MC LUT is used to infer $\mu_{s}^{\prime }\left(\lambda \right)$ from corrected same-wavelength spatial ratios, and a semi-infinite diffusive spatially resolved spectroscopy (SRS) forward model (dipole formulation) [19,39] is swept over candidate $f_{w}$ values to compare modeled and measured ratios. In this work, the diffusion/SRS stage is used to assess directionality and internal consistency (i.e., whether the recovered ${\hat{f}}_{w}$ increases across the phantom series as recipe water fraction increases), rather than to provide a blind, stand-alone estimate of absolute water content for unknown samples. Table 2 summarizes the key notations used to derive the fraction of water in the produced gelatin phantoms.

#### 2.5.1. Notation, Units, and Measured Channels

Two wavelengths are used, λ∈{1450,1650}nm, as well as two source–detector separations, $\rho_{c}$ (close) and $\rho_{f}$ (far):

(4) $$\rho_{c}=0.45\;\mathrm{cm},\;\rho_{f}=0.70\;\mathrm{cm}.$$

For each measurement cycle, the microcontroller samples the conditioned photodetector voltage for each LED state:

(5) $$V_{\lambda ,\rho_{c}}^{\mathrm{on}},\;V_{\lambda ,\rho_{f}}^{\mathrm{on}},\;\lambda \in \left\{1450,1650\right\}\;\mathrm{nm}.$$

An LED-off baseline is recorded immediately before each LED measurement:

(6) $$V_{\lambda ,\rho }^{\mathrm{dark}},\;\rho \in \left\{\rho_{c},\rho_{f}\right\}.$$

#### 2.5.2. Dark Subtraction and Channel-Wise Normalization

Additive offsets and ambient contributions are removed by dark subtraction:

(7) $$S_{\lambda ,\rho }^{\mathrm{raw}}=V_{\lambda ,\rho }^{\mathrm{on}}-V_{\lambda ,\rho }^{\mathrm{dark}},\;\rho \in \left\{\rho_{c},\rho_{f}\right\}.$$

Because the close and far channels use distinct emitters, same-session reference normalization (PTFE) is used to suppress channel-to-channel scale factors:

(8) $$S_{\lambda ,\rho }^{\mathrm{norm}}=\frac{S_{\lambda ,\rho ,\mathrm{sample}}^{\mathrm{raw}}}{S_{\lambda ,\rho ,\mathrm{ref}}^{\mathrm{raw}}}.$$

#### 2.5.3. Corrected Measured Spatial Ratios (PTFE Applied Once)

A corrected same-wavelength spatial ratio is then formed:

(9) $$r_{\lambda }^{\mathrm{corr}}=\frac{S_{\lambda ,\rho_{f}}^{\mathrm{norm}}}{S_{\lambda ,\rho_{c}}^{\mathrm{norm}}}=\frac{S_{\lambda ,\rho_{f},\mathrm{sample}}^{\mathrm{raw}}}{S_{\lambda ,\rho_{c},\mathrm{sample}}^{\mathrm{raw}}}\cdot \frac{S_{\lambda ,\rho_{c},\mathrm{ref}}^{\mathrm{raw}}}{S_{\lambda ,\rho_{f},\mathrm{ref}}^{\mathrm{raw}}}.$$

In what follows, transport-model ratios (MC LUT and diffusion) are matched directly to $r_{\lambda }^{\mathrm{corr}}$; absolute signal magnitudes are not interpreted in this study.

#### 2.5.4. MC LUT Inversion for Reduced Scattering (Ratio Matching in the Corrected Domain)

For each wavelength, the MC LUT provides the probe-matched ratio

(10) $$r_{\lambda }^{\mathrm{MC}}\;\left(\mu_{s,\lambda }^{\prime }\right)=\frac{S_{\lambda ,\rho_{f}}^{\mathrm{MC}}\left(\mu_{s,\lambda }^{\prime }\right)}{S_{\lambda ,\rho_{c}}^{\mathrm{MC}}\left(\mu_{s,\lambda }^{\prime }\right)},$$

where $r_{\lambda }^{\mathrm{MC}}$ is the LUT-predicted ratio and SMC denotes the MC-predicted signal.

Over the inversion-relevant range, $r_{\lambda }^{\mathrm{MC}}\left(\mu_{s}^{\prime }\right)$ is monotonic, enabling one-dimensional inversion. ${\hat{\mu }}_{s,\lambda }^{\prime }$ is computed by interpolation between neighboring LUT points. If two LUT entries $\left(\mu_{1}^{\prime },r_{1}\right)$ and $\left(\mu_{2}^{\prime },r_{2}\right)$ bracket the measurement, log-domain interpolation in the ratio is used:

(11) $${\hat{\mu }}_{s,\lambda }^{\prime }=\mu_{1}^{\prime }+\left(\mu_{2}^{\prime }-\mu_{1}^{\prime }\right)\frac{\ln\;\left(r_{\lambda }^{\mathrm{corr}}\right)-\ln\left(r_{1}\right)}{\ln\left(r_{2}\right)-\ln\left(r_{1}\right)}.$$

where $\mu_{1}^{\prime }$ and $\mu_{2}^{\prime }$ are the two bracketing LUT scattering values (units ${\mathrm{cm}}^{-1}$), $r_{1}$ and $r_{2}$ are the corresponding LUT ratios (unitless), and ln(·) denotes the natural logarithm.

Measurements yielding non-positive normalized signals are flagged as low-SNR and excluded from inversion to avoid undefined logarithms. SNR denotes signal-to-noise ratio.

#### 2.5.5. Absorption Model Parameterized by Water Fraction (Assigned, Not Estimated, in This Study)

After scattering is estimated from the LUT, absorption is parameterized by a candidate bulk water fraction $f_{w}\in \left[0,1\right]$. A general mixture model can be written as

(12) $$\mu_{a,\lambda }\left(f_{w}\right)=\sum_{i}\phi_{i}\left(f_{w}\right)\;\mu_{a,i}\left(\lambda \right),$$

and is approximated here as water dominated over the two-wavelength set:

(13) $$\mu_{a,\lambda }\left(f_{w}\right)\approx f_{w}\;\mu_{a,\mathrm{water}}\left(\lambda \right).$$

Finite LED spectral bandwidth (especially near 1450 nm) and any non-water absorption/effective-medium effects that vary with Intralipid loading are treated as limitations; in this validation, the $\mu_{a,\lambda }$ used for LUT generation/inversion is assigned from nominal $f_{w,\mathrm{true}}$, so any recovered ${\hat{f}}_{w}$ from the forward-model sweep is interpreted as a consistency check rather than blind absorption estimation.

#### 2.5.6. Diffusion/SRS Forward Model

To relate candidate water fraction to expected spatial falloff, we use a semi-infinite diffusion-based dipole model as a parametric signal proxy [19,39]. Using the standard dipole construction with extrapolated boundary conditions [19,39], the predicted intensity (up to an unknown multiplicative coupling constant $C_{\lambda }$) is

(14) $$U_{\lambda }\left(\rho ;f_{w}\right)=\frac{C_{\lambda }}{4\pi D_{\lambda }\left(f_{w}\right)}\left(\frac{e^{-\mu_{\mathrm{eff},\lambda }\left(f_{w}\right)\;r_{1,\lambda }\left(\rho ;f_{w}\right)}}{r_{1,\lambda }\left(\rho ;f_{w}\right)}-\frac{e^{-\mu_{\mathrm{eff},\lambda }\left(f_{w}\right)\;r_{2,\lambda }\left(\rho ;f_{w}\right)}}{r_{2,\lambda }\left(\rho ;f_{w}\right)}\right),$$

where $r_{1,\lambda }$ and $r_{2,\lambda }$ are the source and image-source distances defined by the dipole geometry (computed as in [19,39] using a first-order refractive index n=1.33; full boundary-parameter expressions are provided in the Supplementary Materials).

$U_{\lambda }$ is the diffusion-model fluence-rate proxy and $C_{\lambda }$ is a wavelength-dependent coupling constant. The modeled same-wavelength spatial ratio is then

(15) $$r_{\lambda }^{\mathrm{model}}\left(f_{w}\right)=\frac{U_{\lambda }\left(\rho_{f};f_{w}\right)}{U_{\lambda }\left(\rho_{c};f_{w}\right)},$$

where $r_{\lambda }^{\mathrm{model}}\left(f_{w}\right)$ is the diffusion-model spatial-ratio proxy (unitless) at wavelength λ as a function of candidate water fraction $f_{w}$.

In Equation (14), the unknown coupling factor $C_{\lambda }$ and the prefactor ${\left(4\pi D_{\lambda }\right)}^{-1}$ cancel, so the ratio depends only on separation and the optical-property terms derived from $\mu_{a,\lambda }\left(f_{w}\right)$ and ${\hat{\mu }}_{s,\lambda }^{\prime }$. Because PTFE normalization is already applied once in $r_{\lambda }^{\mathrm{corr}}$ (Equation (9)), no reference term is included inside the transport-model ratio.

#### 2.5.7. Error Function and Water-Fraction Minimization (Consistency-Check Estimate)

Candidate $f_{w}$ values are evaluated by comparing modeled and corrected measured spatial ratios using a log-domain squared error:

(16) $$E\left(f_{w}\right)=\sum_{k=1}^{K}{\left[\ln\;\left(r_{k}^{\mathrm{corr}}\right)-\ln\;\left(r_{k}^{\mathrm{model}}\left(f_{w}\right)\right)\right]}^{2},$$

where $E(f_{w})$ is the scalar (unitless) error assigned to candidate $f_{w}$, *k* indexes the set of ratio constraints included in the consistency-check objective (e.g., wavelengths and/or measurement channels), and *K* is the number of terms used.

A bounded sweep is performed over a physically plausible interval F⊂[0,1], and the final estimate is

(17) $${\hat{f}}_{w}=\arg\min_{f_{w}\in F}E\left(f_{w}\right).$$

where F is the chosen feasible set of candidate water fractions. In this study, ${\hat{f}}_{w}$ is interpreted as a calibration-anchored consistency-check estimate under the absorption assignment and modeling assumptions used; across the phantom series, the primary expectation is that ${\hat{f}}_{w}$ increases monotonically with recipe-defined $f_{w,\mathrm{true}}$ under the stated conditioning. Extension to unknown samples would require coupling the absorption-conditional LUT step to the $f_{w}$ sweep (e.g., a 2D LUT over $\left(\mu_{a},\mu_{s}^{\prime }\right)$ or an iterative scheme), which is outside the validated scope here.

#### 2.5.8. Interpretation Considerations and Failure Modes

Practical failure modes include: (i) phantom non-homogeneity (stratification) that violates the single $\left(\mu_{a},\mu_{s}^{\prime }\right)$ per wavelength assumption; (ii) mismatch between the recipe-defined water-fraction reference and the absorption proxy in Equation (13) (e.g., differing treatment of Intralipid solvent water or mass-fraction-as-volume-fraction approximations); and (iii) joint dependence of spatial ratios on both absorption and scattering, which can degrade estimation accuracy. The initial LUT-based scattering recovery depends on an assigned $\mu_{a}\left(\lambda \right)$. Any inaccuracies in the initial water-fraction assumption can lead to a systematic bias in the $\mu_{s}^{\prime }\left(\lambda \right)$ estimate. Future versions of the algorithm will use 2D lookup tables or iterative methods to estimate $\mu_{a}$ and $\mu_{s}^{\prime }$ together. This will remove the need for recipe-conditioned absorption assignments and allow for true blind recovery.

## 3. Results

### 3.1. Monte Carlo LUT Characterization and Scattering Observables

**Figure 5:**
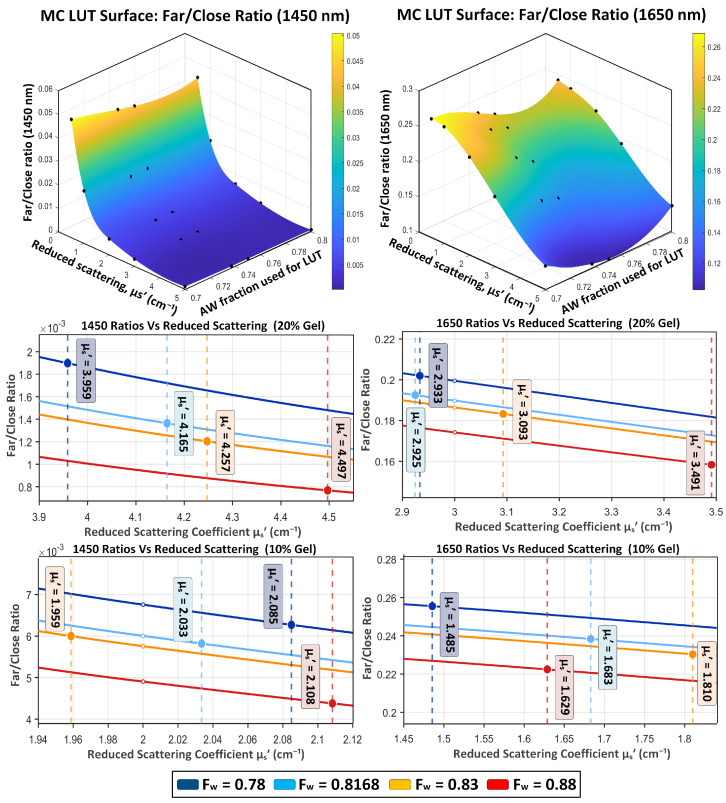
Monte Carlo (MC) lookup–table (LUT) visualization of same-wavelength far/close spatial ratios for gelatin–Intralipid phantoms at 1450 nm (left column) and 1650 nm (right column). Top row: interpolated 3D LUT surfaces of the MC-predicted far/close ratio $r_{\mathrm{MC},\lambda }=S_{\lambda ,\rho_{f}}/S_{\lambda ,\rho_{c}}$ shown versus reduced scattering coefficient $\mu_{s}^{\prime }$ and the added-water (AW) condition used to assign $\mu_{a}\left(\lambda \right)$ during LUT generation; black points indicate the discrete simulated LUT entries. Middle row: zoomed ratio–curve views versus $\mu_{s}^{\prime }$ for the 20% Intralipid dataset. Bottom row: zoomed ratio-curve views versus $\mu_{s}^{\prime }$ for the 10% Intralipid dataset. In each zoomed panel, vertical dashed lines indicate the LUT-inverted ${\hat{\mu }}_{s}^{\prime }$ values for each phantom and filled markers denote the intersection of each inversion line with its corresponding ratio curve. For readability, the 1450 nm ratio panels are displayed with a $\times 10^{3}$ scale factor. Curves/markers are grouped by the formulation-derived reference water fraction $f_{w,\mathrm{true}}$ (color-coded).

Geometry-matched Monte Carlo (MC) simulations were used to generate lookup tables (LUTs) that relate reduced scattering coefficient $\mu_{s}^{\prime }$ to expected diffuse-reflectance observables for the four optical channels (1450 nm and 1650 nm at 4.5 mm and 7 mm source–detector separations). For each phantom water-fraction condition, MC simulations were run using absorption coefficients computed from the formulation-derived bulk water fraction $f_{w,\mathrm{true}}$ (water-dominant absorption parameterization) while sweeping $\mu_{s}^{\prime }$ over the range of interest. The coupling required for unknown samples is described in Section 2.5. Representative log-scale magnitude LUT sweeps are provided in the Supplementary Materials (Figure S2) and are used primarily to verify channel ordering and dynamic-range behavior. Full LUT tables are provided in the Supplementary Materials (Tables S7–S10). For visualization, the 1450 nm and 1650 nm far/close LUTs are also shown as interpolated 3D surfaces over added-water fraction and $\mu_{s}^{\prime }$ in Figure 5.

To isolate a scattering-sensitive observable that is robust to unknown scale factors (LED radiant power, coupling, detector responsivity, and gain), same-wavelength far/close ratios were computed from the LUTs. Figure 5 shows that the far/close ratio at each wavelength is monotonic with $\mu_{s}^{\prime }$, enabling $\mu_{s}^{\prime }$ inversion via interpolation. The relatively small separation between ratio curves across different $f_{w}$ is expected because same-wavelength spatial ratios largely suppress common multiplicative scaling; in this construction, curve-to-curve shifts reflect absorption-dependent transport differences (through the $f_{w}\to \mu_{a}$ assignment) rather than arbitrary LED scaling.

The 1450 nm ratio exhibits a more compressed dynamic range than 1650 nm, consistent with stronger water absorption at 1450 nm suppressing longer photon paths and reducing far/close contrast. In practice, scattering recovery is performed within the $\mu_{s}^{\prime }$ regimes relevant to the experimental phantoms. For clarity, Figure 5 includes zoomed views around the recovered $\mu_{s}^{\prime }$ ranges for the 10% and 20% Intralipid datasets, with inversion markers overlaid.

### 3.2. Water-Fraction Consistency-Check Agreement Analysis

Using the corrected measured spatial ratios and the absorption-conditional MC-derived scattering estimates (via LUT interpolation), a diffusion/SRS forward-model sweep was performed as a calibration-anchored consistency check on eight Intralipid-containing gelatin phantoms (four phantoms at 10% Intralipid stock loading and four phantoms at 20% Intralipid stock loading). In this controlled phantom-validation analysis, $\mu_{a}\left(\lambda \right)$ was parameterized using the recipe-defined nominal $f_{w,\mathrm{true}}$ (via the water-dominant model described in Section 2.5) to evaluate whether the pipeline preserves the nominal ordering and values of $f_{w,\mathrm{true}}$ under absorption-consistent conditions. Each value reported corresponds to a single averaged measurement per phantom under a fixed acquisition configuration.

Across the eight Intralipid-containing phantoms, the recovered $f_{w}$ values preserved the expected ordering with $f_{w,\mathrm{true}}$ and yielded a mean absolute percent error of 1.55% with a maximum absolute percent error of 3.33% (Table 3), conditional on the recipe-defined absorption parameterization. Errors were larger in the 10% Intralipid dataset (mean absolute percent error 2.36%) than in the 20% Intralipid dataset (0.74%). The differences were predominantly negative (SRS underestimation), suggesting that the dominant error component in this dataset is systematic rather than purely random scatter. In addition, the LUT stage produced wavelength-specific scattering estimates in the expected Intralipid regime (Table 3), supporting that the spatial-ratio inversion was operating within a well-conditioned region of the ratio curves.

Although the primary hydration sensitivity is expected near 1450 nm, the 1650 nm band provides a complementary constraint because it lies in a lower-absorption water band and can be less susceptible to modeling mismatch near the steep 1450 nm absorption peak (e.g., finite LED bandwidth and effective $\mu_{a}$). In the two-wavelength formulation (Equation (16)), this secondary band helps regularize the $f_{w}$ sweep by reducing reliance on a single wavelength term and provides additional leverage to distinguish absorption-driven and scattering-driven changes in the measured spatial ratios.

**Figure 6:**
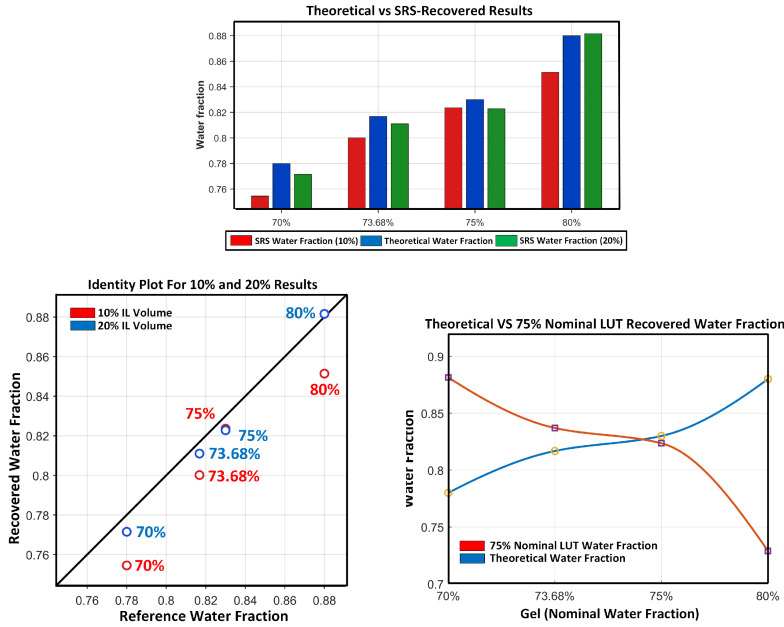
Combined results figure summarizing water-fraction recovery and a fixed-LUT sensitivity check. Top: recovered ${\hat{f}}_{w}$ (SRS) and reference $f_{w,\mathrm{true}}$ grouped by phantom condition (10% and 20% Intralipid stock loading). Bottom Left: identity plot of ${\hat{f}}_{w}$ versus $f_{w,\mathrm{true}}$; the diagonal line indicates y=x (perfect agreement). Bottom Right: “75% nominal” LUT comparison showing theoretical water fraction versus the recovered trend when using a single nominal LUT condition across gels rather than recipe-conditioned LUTs.

Agreement is visualized using an identity plot (Figure 6, top) that compares the recovered water fraction ${\hat{f}}_{w}$ to the formulation-derived reference $f_{w,\mathrm{true}}$, with the y=x line indicating perfect agreement. The identity plot highlights the overall clustering near the diagonal as well as a consistent negative offset across most phantoms. For this dataset (n=8), the overall bias (SRS − reference) was −0.0122 (absolute water-fraction units), with MAE=0.0125 and RMSE=0.0156.

To illustrate sensitivity to LUT selection (i.e., absorption-conditionality of the ratio-to-$\mu_{s}^{\prime }$ mapping), Figure 6 also includes a “75% nominal” comparison (bottom), where a single LUT condition is applied across gels rather than using recipe-conditioned LUTs. This fixed-LUT case visibly deviates from the theoretical trend, motivating coupled or expanded LUT strategies (e.g., 2D LUTs over $\left(\mu_{a},\mu_{s}^{\prime }\right)$ or iterative LUT selection during the $f_{w}$ sweep) for truly unknown samples in the future.

### 3.3. Water-Fraction Repeatability Test

**Figure 7:**
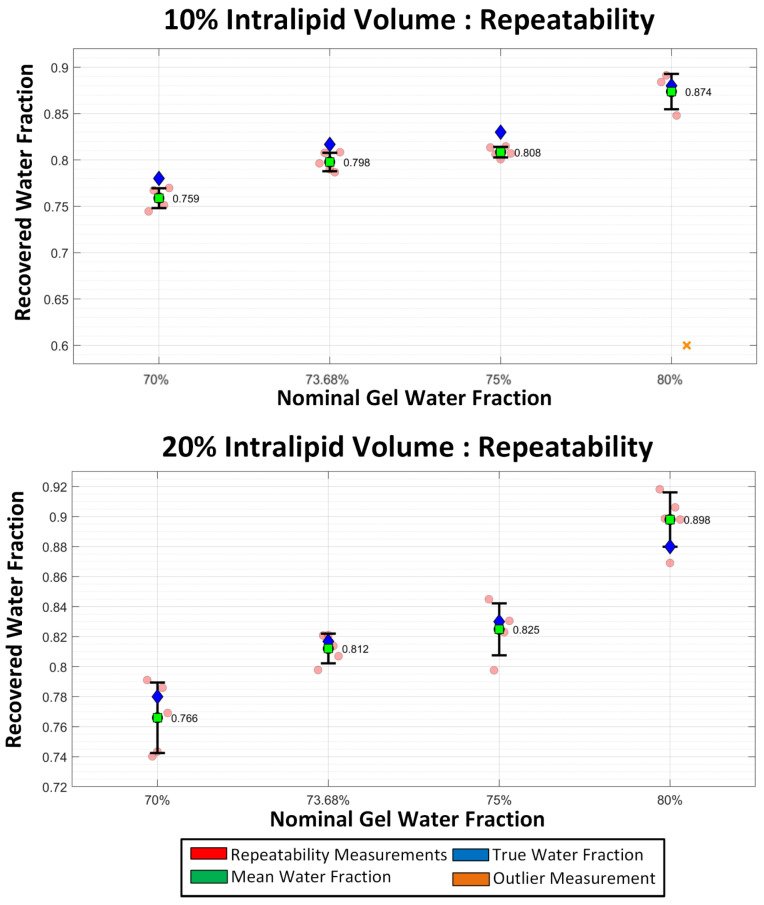
Repeatability of recovered water fraction for gel phantoms with different nominal water content and two scattering conditions. Recovered water fraction obtained from repeated measurements of tissue-mimicking gel phantoms containing 10% (top) and 20% (bottom) Intralipid is shown for nominal gel water fractions of 70%, 73.68%, 75%, and 80%. Red circles represent individual repeatability measurements, green squares denote the mean recovered water fraction, blue diamonds indicate the true (nominal) water fraction used in phantom preparation, and the orange cross indicates an outlier due to experimental error in measurement. Error bars show ±1 standard deviation across measurements. An outlier measurement is indicated by an orange cross. The close clustering of measurements demonstrates good repeatability of the water-fraction inversion across multiple scattering levels.

To evaluate random error and sensitivity to probe positioning, we performed a series of repeated measurements with the probe applied multiple times for both the 10% and 20% Intralipid datasets (Figure 7). In the 10% Intralipid group, the recovered water fractions showed high precision, with mean values of 0.759, 0.798, 0.808, and 0.874 matching the nominal targets. The 20% group also tracked the nominal targets with mean recovered values of 0.766, 0.812, 0.825, and 0.898. These results show that the variance due to positioning is much smaller than the systematic hydration-sensitive shifts seen throughout the series. However, we identified one outlier and excluded it from the final error analysis.

## 4. Discussion

### 4.1. Principal Findings

This study demonstrates a probe-geometry-matched, two-step analysis pipeline for SWIR diffuse reflectance using two wavelengths (1450 nm and 1650 nm) and two source–detector separations (4.5 mm and 7 mm). Probe-specific Monte Carlo (MC) lookup tables (LUTs) map same-wavelength spatial ratios to reduced scattering $\mu_{s}^{\prime }$, and the resulting $\mu_{s}^{\prime }\left(\lambda \right)$ values are used in a semi-infinite diffusive SRS forward model to perform a feasibility study to detect the water fraction in homogeneous media. In this phantom-validation configuration, $\mu_{a}\left(\lambda \right)$ is assigned from the recipe-defined (nominal) phantom water fraction (with stated uncertainty) rather than estimated from reflectance; therefore, recovered ${\hat{f}}_{w}$ values reflect conditional recovery under controlled assumptions rather than blind inference for unknown tissue samples. This approach establishes the intrinsic hydration sensitivity, stability, and internal consistency of the measurement geometry and inversion framework, creating a validated baseline against which future coupled absorption–scattering implementations can be rigorously evaluated.

Across eight Intralipid-containing gelatin phantoms spanning formulation-derived theoretical $f_{w}\approx 0.78$–0.88, recovered water fractions preserved the expected ordering and agreed with theoretical values, with a mean absolute percent error of 1.55% and a maximum absolute percent error of 3.33% (Table 3). The identity-plot agreement showed a consistent negative bias (systematic underestimation) rather than large random scatter (Figure 6, top), indicating stable behavior under fixed acquisition conditions but motivating refinement to reduce offset. The same combined results figure also includes a “75% nominal” fixed-LUT comparison (Figure 6, bottom), which illustrates the sensitivity of recovered trends to LUT selection when absorption is not matched to each phantom condition.

### 4.2. Physical Interpretation of Scattering Observables and Spatial Ratios

The MC LUTs support two practical conclusions for the measurement design. First, channel magnitudes span a wide dynamic range across $\mu_{s}^{\prime }$ and across wavelengths/SDS, so log-scale visualization is useful (see Supplementary Figure S2); the inversion itself relies on ratio observables and compares ratios in the log domain to emphasize multiplicative mismatch and reduce sensitivity to residual scale factors. Second, same-wavelength far/close ratios provide a compact observable for scattering recovery because many session-specific multiplicative factors are reduced by reference normalization and ratio formation. The ratio remains jointly dependent on scattering and absorption through distance-dependent attenuation, but is monotonic in $\mu_{s}^{\prime }$ over the phantom regimes studied, enabling interpolation-based inversion.

Figure 5 provides an additional geometric and conditioning perspective by visualizing the far/close LUTs as interpolated 3D surfaces over the LUT parameter axes (added-water condition used to assign $\mu_{a}\left(\lambda \right)$ and $\mu_{s}^{\prime }$). The smooth surface structure and absence of folds in the operating region indicate that the ratio-to-$\mu_{s}^{\prime }$ mapping is well behaved for these phantom regimes, and the overlaid discrete simulation points verify that the surface interpolation is not introducing obvious artifacts. The corresponding zoomed 2D panels (Figure 5, middle and bottom rows) make explicit where the experimental inversions lie on these surfaces/curves and show that the inverted ${\hat{\mu }}_{s}^{\prime }$ values occur within the monotonic, well-conditioned portion of each ratio curve for both the 10% and 20% Intralipid datasets.

The differences in behavior between 1450 nm and 1650 nm are physically consistent. Stronger water absorption at 1450 nm suppresses longer photon paths, compressing far/close contrast relative to 1650 nm. At 1650 nm, reduced absorption permits a larger contribution of longer paths to the far separation, yielding a larger ratio dynamic range and potentially improved sensitivity to $\mu_{s}^{\prime }$ in the operating regime. Moreover, including both wavelengths provides complementary constraints for downstream consistency-check estimation: 1450 nm offers strong hydration sensitivity near the dominant water band, while 1650 nm lies in a lower-absorption water band and can be less susceptible to modeling mismatch near the steep 1450 nm peak (e.g., finite LED bandwidth and effective $\mu_{a}$). The close separation provides higher SNR superficial sampling, while the far separation increases sensitivity to optical-property changes that affect spatial falloff at the cost of lower magnitude.

The choice of specific 1450 nm and 1650 nm bands focused on creating a compact, wearable design without the complications of a broadband spectrometer. The current two-wavelength setup achieved a Mean Absolute Percent Error (MAPE) of 1.55% in controlled phantoms. Adding more frequency bands could enhance detection accuracy. For example, adding a band around 1200 nm (which has low water absorption) could allow for deeper tissue penetration. On the other hand, a band at 1950 nm (with very high water absorption) could improve sensitivity for superficial measurements. This would help the system better differentiate water from lipid contributions in more varied biological samples.

### 4.3. Limitations and Sources of Systematic Bias

A few factors condition the interpretation of the agreement metrics. First, the diffusion theory provides a computationally efficient parametric sweep, it is known to be approximate at the millimeter-scale source–detector separations and high absorption levels ($\mu_{a}>0.1\mu_{s}^{\prime }$) characteristic of the SWIR bands. In these regimes, standard diffusion models can exhibit significant deviations from the radiative transport equation, typically manifesting as an underestimation of reflectance by 5–15% compared to gold-standard Monte Carlo simulations [22]. By utilizing a hybrid inversion pipeline—where the scattering recovery ($\mu_{s}^{\prime }$) is performed via a probe-geometry-matched MC-LUT—we mitigate the primary geometric and high-absorption transport errors. The subsequent diffusion-based sweep for ${\hat{f}}_{w}$ then operates on these MC-corrected scattering values, reducing the impact of the diffusion approximation on the final water-fraction trend.

Second, the inversion framework is hybrid: probe-geometry-matched Monte Carlo (MC) lookup tables (LUTs) recover $\mu_{s}^{\prime }$, while a semi-infinite diffusion model provides the water-fraction sweep. Diffusion theory is approximate at millimeter-scale source–detector separations in strongly absorbing SWIR bands, and deviations from semi-infinite assumptions (finite phantom thickness, boundary effects, contact variability, and aperture constraints) can introduce systematic offsets.

Third, LUT-based scattering recovery is absorption-conditional. In this validation, $\mu_{a}\left(\lambda \right)$ was assigned from the recipe-defined $f_{w,\mathrm{true}}$ to evaluate internal consistency under controlled conditions. For unknown samples, this conditionality must be removed via coupled strategies, such as a 2D LUT over $\left(\mu_{a},\mu_{s}^{\prime }\right)$ or iterative absorption–scattering updates during the $f_{w}$ sweep.

The fixed-LUT comparison shown in Figure 6 directly illustrates this dependence: applying a single nominal absorption condition across gels disrupts ordering and magnitude agreement, confirming that far/close spatial ratios depend jointly on $\mu_{a}$ and $\mu_{s}^{\prime }$.

Despite these constraints, the results demonstrate that the compact probe architecture preserves monotonic hydration trends and achieves < 3.33% maximum absolute error under absorption-consistent conditions, establishing the geometric and transport-model fidelity necessary for extension to blind inversion frameworks.

The repeatability study, Figure 7, demonstrates that while contact pressure and probe orientation introduce measurable variance, the system’s precision remains sufficient to resolve hydration changes within the validated step-sizes.

### 4.4. Implications and Next Steps

These phantom-validation results demonstrate that a compact SWIR reflectance probe produces repeatable, monotonic spatial-ratio signatures with respect to recipe-defined water fractions in gelatin–Intralipid media under absorption-parameterized conditions. The study therefore establishes hydration-sensitive signal behavior and inversion self-consistency in a controlled optical setting, rather than serving as a blind estimator of tissue water fraction. The findings motivate targeted refinements to improve SNR, tighten control of effective source–detector separation, and strengthen correspondence between hardware and Monte Carlo (MC) models.

For translation to unknown samples, two modeling upgrades are indicated: (i) generate LUTs over a 2D grid of $\left(\mu_{a},\mu_{s}^{\prime }\right)$ (or equivalently $\left(f_{w},\mu_{s}^{\prime }\right)$ with an explicit absorption model) to enable coupled scattering inversion during the sweep, and/or (ii) implement an iterative scheme in which a candidate $f_{w}$ defines $\mu_{a}\left(\lambda \right)$, selects the appropriate LUT slice, updates $\mu_{s}^{\prime }\left(\lambda \right)$, and repeats to convergence. Either strategy removes reliance on recipe-conditioned absorption and enables principled blind recovery with uncertainty quantification.

On the hardware side, closer alignment between the MC source model and physical emission geometry is required. Because discrete LEDs launch from finite standoff with angular sensitivity, an aperture-defined effective source is recommended: combine same-wavelength LEDs into a short mixing element (e.g., light pipe or integrating cavity) terminating at a fixed exit aperture with a precisely defined offset. This approach increases photon budget and SNR while providing a well-defined MC boundary condition. Similarly, adding a defined collection optic (e.g., light guide or concentrator) can stabilize acceptance geometry and improve repeatability; such changes can be incorporated into future LUT generation.

Finally, mismatched package dimensions between the 1450 nm and 1650 nm LEDs introduced small but systematic differences in effective source position and launch conditions that are difficult to fully capture in simplified MC models. A clear next step is to use wavelength-matched LEDs in identical, smaller-footprint, higher-radiance packages so both channels share a common mechanical datum, improving geometric consistency and optical power simultaneously.

## Figures and Tables

**Table 1:** Component masses used to prepare 15.0 g gelatin phantoms at two Intralipid^®^ 20% stock loadings (10% and 20% of total batch mass). “Added-water (AW)” labels indicate the fraction of added distilled water by total batch mass. “True water content” accounts for water contained in Intralipid^®^ 20% stock (assumed 80% water by mass): $f_{w,\mathrm{true}}=\left(m_{\mathrm{water}}+0.8\;m_{\mathrm{IL}}\right)/m_{\mathrm{total}}$.

Phantom	Intralipid Stock Loading (% of Batch Mass)	Water (g)	Gelatin (g)	Intralipid^®^ 20% (g)	True Water Content (%)
70% AW	10%	10.50	3.000	1.50	78.00
73.68% AW	10%	11.052	2.448	1.50	81.68
75% AW	10%	11.25	2.250	1.50	83.00
80% AW	10%	12.00	1.500	1.50	88.00
62% AW	20%	9.300	2.700	3.00	78.00
65.68% AW	20%	9.852	2.148	3.00	81.68
67% AW	20%	10.050	1.950	3.00	83.00
72% AW	20%	10.800	1.200	3.00	88.00

**Table 2:** Key symbols and units used throughout the manuscript.

Symbol	Definition	Units
$\mu_{a}$	Absorption coefficient	cm^−1^
$\mu_{a,\lambda }\left(f_{w}\right)$	Absorption coefficient at wavelength λ parameterized by $f_{w}$	cm^−1^
$\mu_{a,\mathrm{water}}\left(\lambda \right)$	Absorption coefficient of pure water at wavelength λ	cm^−1^
$\mu_{s}$	Scattering coefficient	cm^−1^
$\mu_{s}^{\prime }$	Reduced scattering coefficient ($\mu_{s}\left(1-g\right)$)	cm^−1^
${\hat{\mu }}_{s,\lambda }^{\prime }$	LUT-inverted reduced scattering coefficient at wavelength λ	cm^−1^
$\mu_{\mathrm{eff}}$	Effective attenuation coefficient	cm^−1^
*g*	Anisotropy factor	–
$\rho_{c}$	Close source–detector separation	cm
$\rho_{f}$	Far source–detector separation	cm
$f_{w}$	Candidate bulk water fraction	–
$f_{w,\mathrm{true}}$	Recipe-defined nominal water fraction	–
$\phi_{i}\left(f_{w}\right)$	Effective fraction of constituent *i* in absorption mixture model	–
$V_{\lambda ,\rho }^{\mathrm{on}}$	Measured photodetector voltage with LED on	V
$V_{\lambda ,\rho }^{\mathrm{dark}}$	Measured photodetector voltage with LED off (dark baseline)	V
$S_{\lambda ,\rho }^{\mathrm{raw}}$	Dark-subtracted signal	V
$S_{\lambda ,\rho }^{\mathrm{norm}}$	Reference (PTFE)-normalized signal	–
$S_{\lambda ,\rho ,\mathrm{ref}}^{\mathrm{raw}}$	Dark-subtracted signal measured on the PTFE reference	–
$S_{\lambda ,\rho }^{\mathrm{MC}}\left(\mu_{s,\lambda }^{\prime }\right)$	MC-predicted detected signal at wavelength λ and separation ρ	–
$r_{\lambda }^{\mathrm{corr}}$	Corrected measured far/close spatial ratio	–
$r_{\lambda }^{\mathrm{MC}}$	Monte Carlo LUT-predicted spatial ratio	–
$r_{\lambda }^{\mathrm{model}}$	Diffusion-model spatial ratio	–
$U_{\lambda }\left(\rho ;f_{w}\right)$	Diffusion-model fluence-rate proxy at separation ρ	a.u.
$C_{\lambda }$	Wavelength-dependent coupling constant	–

**Table 3:** Comparison of formulation-derived reference water fraction and SRS-recovered water fraction for gelatin–Intralipid phantoms. Gel IDs report the added-water fraction used during formulation (excluding solvent water contained in Intralipid stock). For the 20% group, added-water fractions are adjusted so that the reference $f_{w,\mathrm{true}}$ matches the corresponding 10% targets (Table 1). Absolute percent error is computed as $\left|{\hat{f}}_{w}-f_{w,\mathrm{true}}\right|/f_{w,\mathrm{true}}\times 100$.

Gel ID	IL (wt%)	${\hat{\mu }}_{s,1450}^{\prime }$	${\hat{\mu }}_{s,1650}^{\prime }$	$f_{w,\mathrm{true}}$	${\hat{f}}_{w}$	Abs. Error (%)
10% Intralipid						
AW70	10	2.085	1.485	0.780	0.754	3.333
AW73.68	10	2.033	1.683	0.817	0.800	2.081
AW75	10	1.959	1.810	0.830	0.824	0.723
AW80	10	2.108	1.629	0.880	0.851	3.295
20% Intralipid						
AW62	20	3.959	2.933	0.780	0.771	1.154
AW65.68	20	4.165	2.925	0.817	0.811	0.734
AW67	20	4.247	3.093	0.830	0.823	0.843
AW72	20	4.497	3.491	0.880	0.882	0.227

## Data Availability

The data presented in this study are available on request from the corresponding author. The data are not publicly available due to privacy.

## Supplementary Materials

The following supporting information can be downloaded at: https://www.mdpi.com/article/10.3390/s26072020/s1.
