# Supplementary material for: Development and Phantom Validation of a Small-Form-Factor SWIR Emitter Probe for Hydration-Sensitive Spatial-Ratio Measurements in Gelatin–Intralipid Phantoms
Source: Sensors (Basel). 2026 Mar 24;26(7):2020. doi: 10.3390/s26072020 (PMC13074628; doi:10.3390/s26072020)
Supplement: Supplementary file 1 [file sensors-26-02020-s001.zip › sensors-4207761-supplementary.pdf]

Supplementary Materials for: Development and Phantom Validation of a Small-Form-Factor SWIR Emitter Probe for Hydration-Sensitive Spatial-Ratio Measurements in Gelatin-Intralipid Phantoms

IMMEDIATE

This supplementary document provides expanded experimental and modeling details to support the primary manuscript, including: (S1) representative gelatin phantom composition context, full recipe tables (percent and mass for 15 mL batches), and stepwise preparation photographs; (S2) acquisition chain details (channel-to-LED mapping, LED drive, timing, averaging) and preprocessing equations including PTFE session normalization; (S3) Monte Carlo (MC) configuration details and full lookup tables (LUTs) used for reduced-scattering inversion; and (S4) the complete diffusion / spatially resolved spectroscopy (SRS) forward model used for the calibration-anchored water-fraction consistency-check.

1. SUPPLEMENTARY CONTENTS

This document contains the extended technical details and full tables that were moved out of the shortened manuscript for page-length reduction. Key items include Table S1 (context phantom compositions), Table S2–S3 (recipes used in this study, percent and mass), Table S4 (channel-to-LED mapping and SDS), Table S6 (acquisition/averaging timing), Table S7 (MC settings extracted from `IdealSimDecember.m`), Fig. S1 (preparation photo sequence), Fig. S2 (MC LUT channel trends), and Tables S8–S11 (full LUTs for the shown gel conditions).

2. S1. PHANTOM COMPOSITION CONTEXT AND PREPARATION

A. Representative phantom composition context (adapted from Jonasson et al.)

Table S1 provides representative gelatin phantom compositions adapted from Jonasson et al. for *context* (not the exact formulations measured in this study). The purpose of this table is to illustrate typical gelatin/water/Intralipid-style phantom ranges used to emulate hydration and edema-like variation in optical studies.

Table S1. Representative gelatin phantom compositions adapted from Jonasson et al. for context (not the specific formulations measured in this study). Total water fraction assumes Intralipid® 20% is 80% water by mass.

Phantom	Water (%)	Gelatin (%)	Intralipid® 20% (%)	Total water fraction
1	89	1	10	0.97
2	80	10	10	0.88
3	60	30	10	0.68
4	40	50	10	0.48

B. Materials and batch size used in this study

All phantoms were prepared as 15 mL batches to fit the 3D-printed molds (≈ 15 g total mass, assuming 1 mL ≈ 1 g). Gelatin was bovine gelatin (250 Bloom; Bulkfoods). Intralipid® was 20 %

stock (MilliporeSigma/Sigma-Aldrich).

B.1. Naming convention and recipe calculation

Gel identifiers use the shorthand **AWxx** to denote the *added-water* percentage (by mass) in the recipe prior to including Intralipid® stock, with the gelatin fraction set by closure:

$$\text{Gelatin}(\%) = 100 - \text{Water}(\%) - \text{IL}(\%). \tag{S1}$$

IL10 and IL20 indicate 10% and 20% Intralipid® 20% stock loadings, respectively. The theoretical total water fraction used throughout the manuscript is computed assuming IL20 stock is 80% water by mass:

$$f_{w,\text{true}} = \frac{\text{Water}(\%) + 0.8\,\text{IL}(\%)}{100}. \tag{S2}$$

For the **IL20-equivalent** recipes, $\text{IL}(\%) = 20$ is fixed and Water(%) is selected to match the corresponding IL10 gel's $f_{w,\text{true}}$:

$$\text{Water}_{\text{IL20eq}}(\%) = 100\, f_{w,\text{target}} - 0.8 \times 20, \qquad \text{Gelatin}_{\text{IL20eq}}(\%) = 100 - \text{Water}_{\text{IL20eq}}(\%) - 20. \tag{S3}$$

All batches were prepared by mass; Table S3 provides weigh-by-gram values for 15 mL (≈ 15 g) batches, which can be scaled linearly for other batch volumes.

Table S2. Gel phantom recipes used in this study following a Jonasson-style gelatin–water–Intralipid formulation. Percentages are recipe fractions (summing to 100%). Total water fraction assumes Intralipid® 20% stock is 80% water: $f_{w,\text{true}} = (\text{Water} + 0.8\,\text{IL})/100$. For IL20 recipes, the water fraction is adjusted so that $f_{w,\text{true}}$ matches the corresponding IL10 gel.

Gel ID	Water (%)	Gelatin (%)	IL% stock (%)	$f_{w,\text{true}}$
**No Intralipid control (IL0)**				
AW78 + IL0 (control)	78.00	22.00	0.00	0.7800
**10% Intralipid® 20% stock (IL10)**				
AW70 + IL10	70.00	20.00	10.00	0.7800
AW73.68 + IL10	73.68	16.32	10.00	0.8168
AW75 + IL10	75.00	15.00	10.00	0.8300
AW80 + IL10	80.00	10.00	10.00	0.8800
**20% Intralipid® 20% stock (IL20; water adjusted to match $f_{w,\text{true}}$)**				
AW70-equivalent + IL20	62.00	18.00	20.00	0.7800
AW73.68-equivalent + IL20	65.68	14.32	20.00	0.8168
AW75-equivalent + IL20	67.00	13.00	20.00	0.8300
AW80-equivalent + IL20	72.00	8.00	20.00	0.8800

Table S3. Component masses for 15 mL batches (approximately 15 g total mass). Masses are computed as $(\%/100) \times 15\,\text{g}$. This is an operational recipe table (weigh-by-mass) and assumes water and IL stock densities are approximately 1 g/mL.

Gel ID	Water (g)	Gelatin (g)	IL% stock (g)	$f_{w,\text{true}}$
AW78 + IL0 (control)	11.70	3.30	0.00	0.7800
AW70 + IL10	10.50	3.00	1.50	0.7800
AW73.68 + IL10	11.052	2.448	1.50	0.8168
AW75 + IL10	11.25	2.25	1.50	0.8300
AW80 + IL10	12.00	1.50	1.50	0.8800
AW70-equivalent + IL20	9.30	2.70	3.00	0.7800
AW73.68-equivalent + IL20	9.852	2.148	3.00	0.8168
AW75-equivalent + IL20	10.05	1.95	3.00	0.8300
AW80-equivalent + IL20	10.80	1.20	3.00	0.8800

B.2. Step-by-step preparation protocol (Jonasson-style)

Phantoms were prepared following a Jonasson-style gelatin–water–Intralipid workflow adapted for small batches:

1. **Weighing:** Water, gelatin powder, and (when applicable) IL20 stock were weighed by mass according to Tables S2–S3.
2. **Blooming:** Gelatin was sprinkled onto the weighed water and allowed to bloom for 5 minutes without stirring.
3. **Dissolution/mixing:** The mixture was stirred (e.g., magnetic stirrer) until visually homogeneous. Heating was used as needed to fully dissolve gelatin while avoiding boiling and excessive bubble formation (45-55 Celcius).
4. **Intralipid addition:** When applicable, IL20 stock was added after gelatin dissolution while continuing stirring to maintain homogeneity. The mixture was mixed until no visible streaks remained.
5. **Degassing:** The solution was vacuum degassed until macroscopic bubbles were removed.
6. **Molding/setting:** The degassed solution was poured into 15 mL 3D-printed molds and allowed to set.
7. **Measurement timing:** Measurements were taken after gels were fully set; the same mold geometry was used for PTFE reference stacks to match probe contact conditions.

This protocol is sufficient to reproduce the reported compositions and measurement geometry; future work will log temperature/time parameters explicitly to improve inter-lab reproducibility.

C. Preparation photo sequence

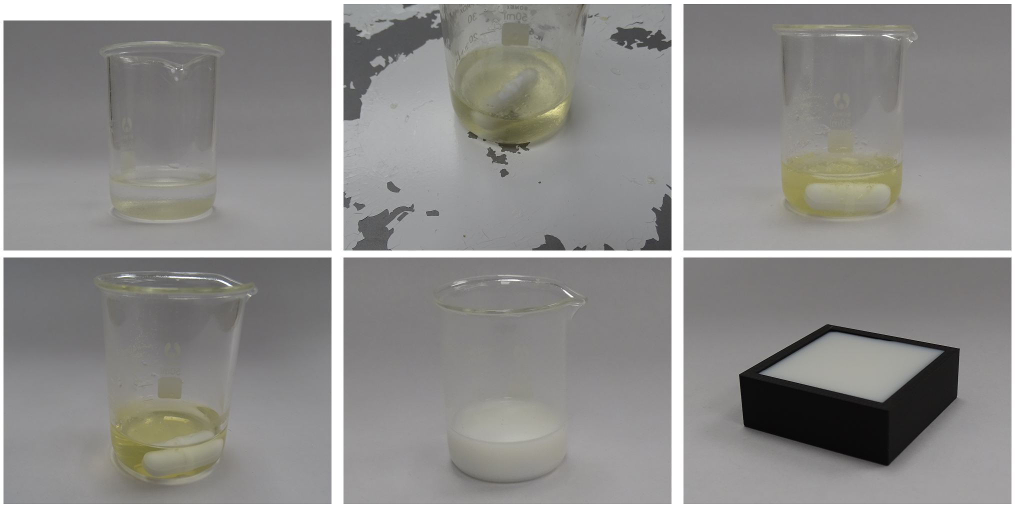

Fig. S1. Gel phantom preparation sequence (gelatin–water mixing through Intralipid addition and degassing). Top row (left to right): blooming, start of mixing, and after 5 min at 500 rpm. Bottom row (left to right): visually homogeneous solution prior to Intralipid addition, mixture after adding Intralipid® 20% stock, and mixture after vacuum degassing.

3. S2. ACQUISITION CHAIN AND PREPROCESSING (EXPANDED)

A. Channel mapping and probe geometry

The device records four channels per measurement cycle: two wavelengths (1,450 nm, 1,650 nm) at two source–detector separations (SDS). The mapping used in this study is given in Table S4.

Table S4. Channel-to-LED mapping and source–detector separation (SDS) used for all reported measurements.

Channel	Wavelength (nm)	SDS (mm)	Label
CH1	1450	4.5	1450-close
CH2	1650	7.0	1650-far
CH3	1450	7.0	1450-far
CH4	1650	4.5	1650-close

For modeling and inversion, separations are expressed in centimeters:

$$\rho_c = 0.45 \text{ cm}, \qquad \rho_f = 0.70 \text{ cm}. \tag{S4}$$

B. LED drive, timing, and averaging

All LEDs are driven at a constant current of 45 mA. Each reported channel value is produced by averaging 50 ADC samples for that LED state. A typical gel session consists of ~ 100 reported measurement cycles, with a 50 ms settling time after each LED switching event before sampling. The average, measurements their standard deviation, and the Coefficient of Variation are reported in Table S5.

Table S5. Statistical summary of raw signal intensities including mean, standard deviation, and coefficient of variation (CV) for gelatin-Intralipid phantoms, with the overall average CV reported at the bottom.

IL Conc.	Gel	1450 nm (4.5 mm)			1650 nm (7.0 mm)			1450 nm (7.0 mm)			1650 nm (4.5 mm)		
	Category	Avg	Stdev	CV	Avg	Stdev	CV	Avg	Stdev	CV	Avg	Stdev	CV
10%	70% AW	0.6050	0.0127	2.10%	0.1599	0.0054	3.37%	0.0407	0.0013	3.08%	0.8426	0.0200	2.38%
	0.7368% AW	0.5851	0.0326	5.57%	0.1339	0.0071	5.30%	0.0371	0.0017	4.57%	0.7347	0.0370	5.03%
	0.75 % AW	0.5176	0.0150	2.89%	0.1297	0.0041	3.13%	0.0422	0.0014	3.27%	0.6918	0.0260	3.76%
	0.8 % AW	0.2098	0.0033	1.55%	0.1014	0.0065	6.45%	0.0367	0.0023	6.15%	0.5301	0.0207	3.91%
20%	70% AW	0.9646	0.0160	1.66%	0.1623	0.0036	2.21%	0.0382	0.0006	1.57%	1.0723	0.0111	1.04%
	0.7368% AW	1.1692	0.0342	2.93%	0.1701	0.0070	4.09%	0.0391	0.0009	2.28%	1.1826	0.0242	2.04%
	0.75% AW	0.7756	0.0167	2.15%	0.1462	0.0108	7.40%	0.0407	0.0008	1.96%	1.0060	0.0285	2.84%
	0.8% AW	1.1422	0.0331	2.90%	0.1480	0.0009	0.59%	0.0418	0.0013	3.00%	1.1625	0.0053	0.46%
**Average CV**								**3.18%**					

The multiplex order is one LED at a time (sequential channel acquisition).

Table S6. Acquisition parameters used during gel measurements.

Parameter	Value
Microcontroller	Arduino Nano 33 (ADC input: A0)
LED drive current (all channels)	45 mA
Settling time after LED state change	50 ms
ADC samples averaged per reported channel value	50
Reported measurement cycles per gel session	~ 100
Reported output per cycle	4 channels (Table S4)
Notes	Each session yields $\sim 100 \times 4 = 400$ reported channel values; each reported value is an average of 50 ADC samples.

B.1. *Per-session averaging and reported values*

For each gel session, approximately $\sim$ 100 measurement cycles were recorded. Within each cycle, each channel value is the mean of 50 ADC samples (Table S6). Unless otherwise stated, the reported channel magnitudes used for ratios and inversion were computed as the *mean across cycles* after dark subtraction and PTFE normalization (Eqs. S9–S11). Cycle-to-cycle variability (standard deviation across the $\sim$ 100 cycles) provides a within-session noise estimate; this work primarily uses the mean value per session for LUT inversion and SRS consistency checks.

C. PTFE reference acquisition for session normalization

A PTFE reference was measured each session by stacking several thin PTFE slabs in the same 3D-printed mold used for gels, to approximate the same probe contact geometry. For each PTFE stack configuration, $\sim$ 100 measurement cycles were acquired and used to compute the session reference term in Eq. S10. This procedure anchors session-to-session normalization while retaining spatial ratio sensitivity.

D. Analog scaling and filtering

The photodetector output is scaled to the microcontroller ADC input range (0–3.3 V) using a resistive divider:

$$V_{\text{ADC,in}} = V_{\text{PD}} \cdot \frac{100}{230 + 100} \approx 0.303\, V_{\text{PD}}. \tag{S5}$$

A low-pass stage suppresses high-frequency noise. For a 2-pole Sallen–Key form:

$$f_0 \approx \frac{1}{2\pi\sqrt{R_1 R_2 C_1 C_2}} \approx 100\,\text{Hz}. \tag{S6}$$

E. Dark subtraction and session normalization

For each LED state, sampled voltages are denoted

$$V^{\text{on}}_{\lambda,\rho_c}, \qquad V^{\text{on}}_{\lambda,\rho_f}, \qquad \lambda \in \{1450, 1650\}\,\text{nm}. \tag{S7}$$

An LED-off (dark) voltage is recorded using the same analog and sampling settings:

$$V^{\text{dark}}_{\lambda,\rho}, \qquad \rho \in \{\rho_c, \rho_f\}. \tag{S8}$$

Dark-subtracted signal:

$$S^{\text{raw}}_{\lambda,\rho} = V^{\text{on}}_{\lambda,\rho} - V^{\text{dark}}_{\lambda,\rho}. \tag{S9}$$

Session normalization (e.g., PTFE reference):

$$S^{\text{norm}}_{\lambda,\rho} = \frac{S^{\text{raw}}_{\lambda,\rho,\text{sample}}}{S^{\text{raw}}_{\lambda,\rho,\text{ref}}}. \tag{S10}$$

Corrected spatial ratio:

$$r^{\text{corr}}_{\lambda} = \frac{S^{\text{norm}}_{\lambda,\rho_f}}{S^{\text{norm}}_{\lambda,\rho_c}} = \frac{S^{\text{raw}}_{\lambda,\rho_f,\text{sample}}}{S^{\text{raw}}_{\lambda,\rho_c,\text{sample}}} \cdot \frac{S^{\text{raw}}_{\lambda,\rho_c,\text{ref}}}{S^{\text{raw}}_{\lambda,\rho_f,\text{ref}}}. \tag{S11}$$

4. S3. MONTE CARLO LUT GENERATION AND SCATTERING INVERSION

A. MC configuration summary (from `MCSimPaper.m`)

Table S7 summarizes key MCmatlab settings used to generate LUTs, extracted from the provided script `IdealSimDecember.m`.

Table S7. Monte Carlo (MCmatlab) parameter summary extracted from `IdealSimDecember.m`.

Parameter	Value in script
Domain size (cm)	$3.5 \times 3.5 \times 1.2$
Grid size (voxels)	$101 \times 101 \times 150$
Voxel size (cm)	$\Delta x \approx 3.5/101$, $\Delta z \approx 1.2/150$
Boundary type	1 (Fresnel reflection enabled)
Matched interfaces	true
Refractive index (medium)	$n = 1.33$
Anisotropy	$g = 0.9$
Source type	Finite Area LED
Source focus depth (cm)	$z_{\text{focus}} = 0.03 + 10^{-4}$
Collector diameter (cm)	0.4
Collector field size (cm)	0.5
Collector NA	0.22
Collector angular resolution	50
Collector focal length (cm)	0.1
SDS list in script (cm)	$[0.45,\ 0.70]$
Photon budget (base)	$N_{\text{base}} = 2 \times 10^7$
Photon budget (cap)	$N_{\text{cap}} = 2 \times 10^9$
Target detected photons	$N_{\text{det,target}} = 1000$ (rerun scaling)
Warn threshold	$N_{\text{det,warn}} = 500$
Max reruns	5
Max scale factor per rerun	20
μ_s' sweep (cm^{-1})	0 to 10 in steps of 0.1 (with $\epsilon = 10^{-4}$)
Absorption model	$\mu_a(\lambda) = f_w \cdot \mu_{a,\text{water}}(\lambda)$
$\mu_{a,\text{water}}(1450)$ (cm^{-1})	28.8
$\mu_{a,\text{water}}(1650)$ (cm^{-1})	5.7

A.1. Absorption-conditioning of LUTs and interpretation

The MC LUTs are *absorption-conditional*: for each gel condition, $\mu_a(\lambda)$ is fixed using the recipe-defined $f_{w,\text{true}}$ and the water absorption coefficients (Hale & Querry), and $\mu_s'(\lambda)$ is swept to tabulate predicted channel magnitudes and spatial ratios. Accordingly, the LUT inversion in Eq. S13 estimates $\hat{\mu}_s'$ under the stated absorption model rather than performing blind recovery of both μ_a and μ_s' from reflectance. For each condition, the underlying Monte Carlo observable is the detected-photon fraction $p_{\text{det}} = N_{\text{det}}/N$, where N is the number of launched photons and N_{det} is the number collected by the probe-emulated detector acceptance. When p_{det} is very small (low N_{det}), LUT ratios can become noisy ("photon starvation"), so the script conditionally increases photon budgets using the $N_{\text{det,target}}$ and $N_{\text{det,warn}}$ thresholds listed in Table S7. Zero entries in the LUT tables indicate that no photons were detected in that channel under the available photon budget at that configuration (i.e., below simulation resolution), not that the physical signal is exactly zero.

B. LUT ratio definition and inversion

For each λ, define the MC-predicted spatial ratio

$$r_\lambda^{\mathrm{MC}}\left(\mu'_{s,\lambda}\right) = \frac{S^{\mathrm{MC}}_{\lambda,\rho_f}(\mu'_{s,\lambda})}{S^{\mathrm{MC}}_{\lambda,\rho_c}(\mu'_{s,\lambda})}. \tag{S12}$$

The scattering estimate $\hat{\mu}'_{s,\lambda}$ is obtained by matching

$$r_\lambda^{\mathrm{MC}}\left(\hat{\mu}'_{s,\lambda}\right) = r_\lambda^{\mathrm{corr}}. \tag{S13}$$

Using log-domain linear interpolation between LUT bracketing points (μ'_1, r_1) and (μ'_2, r_2):

$$\hat{\mu}'_{s,\lambda} = \mu'_1 + (\mu'_2 - \mu'_1)\,\frac{\ln(r_\lambda^{\mathrm{corr}}) - \ln(r_1)}{\ln(r_2) - \ln(r_1)}. \tag{S14}$$

C. MC channel trends (log-scale panels)

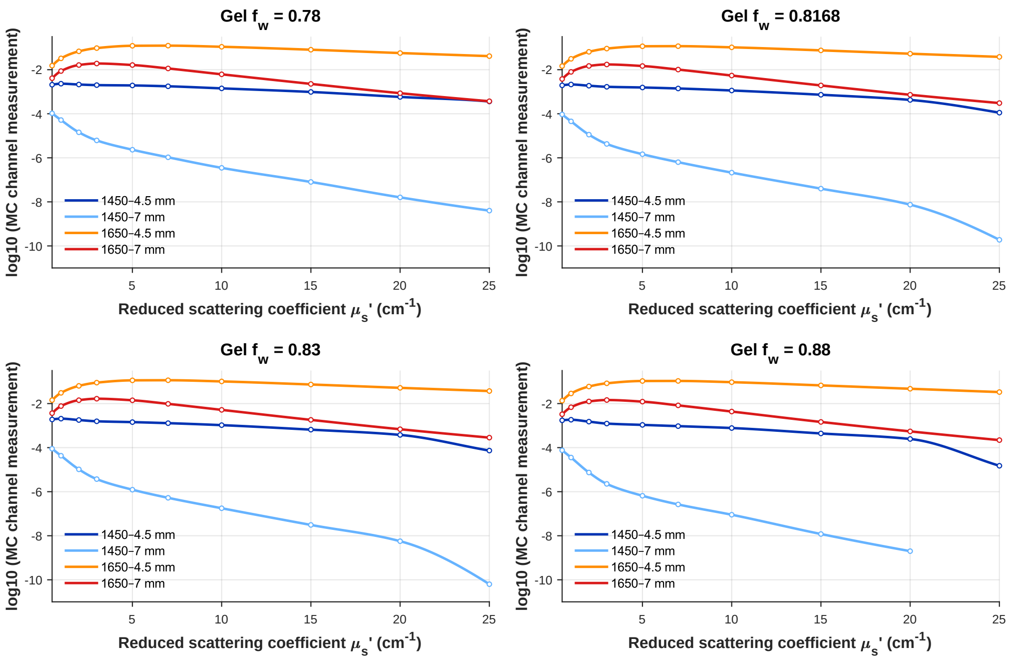

Fig. S2. Monte Carlo LUT channel measurements plotted as $\log_{10}(\text{measurement})$ versus reduced scattering coefficient μ'_s. Panels are ordered from lowest to highest f_w (top-left to bottom-right). Each panel overlays the four simulated channels (1450 nm and 1650 nm at 4.5 mm and 7 mm SDS).

D. Full LUT tables

Note: Zeros in LUT entries indicate that no photons were detected in that channel at the simulated photon count (i.e., below the simulation's resolution at that configuration).

Table S8. Lookup table (LUT) for Gel ID AW70 + IL10% (added-water 70%, theoretical $f_w = 0.7800$), showing simulated channel magnitudes at 4.5 mm and 7 mm SDS and derived far/close ratios versus μ'_s.

μ'_s (cm^{-1})	1450 nm 4.5 mm	1450 nm 7 mm	1650 nm 4.5 mm	1650 nm 7 mm	Far/Close 1450	Far/Close 1650
0.5	0.002078907	0.00010469	0.01524558	0.0040958	0.050358193	0.268654915
1	0.002313882	0.000051822	0.03266623	0.0086768	0.022396129	0.265619877

μ_s' (cm^{-1})	1450 nm 4.5 mm	1450 nm 7 mm	1650 nm 4.5 mm	1650 nm 7 mm	Far/Close 1450	Far/Close 1650
2	0.002122957	0.000014348	0.06817515	0.0162878	0.006758498	0.238911099
3	0.001995686	0.000006162	0.09559348	0.0190692	0.00308766	0.199482224
5	0.001926438	0.000002322	0.1217046	0.016297	0.001205333	0.133906196
7	0.001763839	0.000001056	0.1234642	0.0113844	0.000598694	0.092208106
10	0.001420242	0.00000035	0.1097012	0.0061718	0.000246437	0.056260096
15	0.000976029	0.00000008	0.0807917	0.0022654	0.000081965	0.028040009
20	0.000580391	0.000000016	0.05703168	0.0008574	0.000027568	0.01503375
25	0.000360147	0.000000004	0.04132461	0.0003692	0.000011107	0.008934144
30	0.000016399	0	0.03024104	0.0001664	0	0.005502456

Table S9. Lookup table (LUT) for Gel ID AW73.68 + IL10% (added-water 73.68%, theoretical $f_w = 0.8168$), showing simulated channel magnitudes at 4.5 mm and 7 mm SDS and the derived far/close ratios versus μ_s'.

μ_s' (cm^{-1})	1450 nm 4.5 mm	1450 nm 7 mm	1650 nm 4.5 mm	1650 nm 7 mm	Far/Close 1450	Far/Close 1650
0.5	0.002106602	0.00009591	0.01336903	0.0033014	0.045525751	0.24687062
1	0.002238513	0.000043645	0.02891577	0.0066742	0.019497233	0.230835328
2	0.001919523	0.000010647	0.05632331	0.0116882	0.005546328	0.207539538
3	0.00180896	0.00000414	0.07407571	0.0120904	0.002288108	0.163245514
5	0.001735494	0.000001568	0.0856235	0.0091422	0.000903583	0.10676824
7	0.001566012	0.000000693	0.08163102	0.0056648	0.000442433	0.069393226
10	0.001215058	0.000000249	0.06840188	0.0029674	0.000204926	0.043382403
15	0.000805317	0.000000054	0.04808577	0.0010522	0.000067054	0.021879429
20	0.000464621	0.000000014	0.0333464	0.0003934	0.000030133	0.011795838
25	0.000287656	0.000000004	0.02399152	0.0001654	0.000013907	0.006894255
30	0.000166846	0.000000001	0.01746416	0.0000732	0.000005994	0.004192896

Table S10. Lookup table (LUT) for Gel ID AW75 + IL10% (added-water 75%, theoretical $f_w = 0.8300$), showing simulated channel magnitudes at 4.5 mm and 7 mm SDS and the derived far/close ratios versus μ_s'.

μ_s' (cm^{-1})	1450 nm 4.5 mm	1450 nm 7 mm	1650 nm 4.5 mm	1650 nm 7 mm	Far/Close 1450	Far/Close 1650
0.5	0.001895341	0.000083037	0.01194799	0.0027994	0.043814009	0.234286757
1	0.001870866	0.000036889	0.02527562	0.0056294	0.019720053	0.222700936
2	0.001598497	0.000009235	0.04959039	0.0094972	0.005776845	0.191512016
3	0.001475778	0.000003502	0.06303093	0.009828	0.002372707	0.155914263
5	0.001410131	0.000001275	0.07152957	0.00736	0.00090423	0.102902383
7	0.001255523	0.00000055	0.0670413	0.0043578	0.000438078	0.065010063
10	0.000944484	0.000000197	0.05558915	0.0022668	0.00020857	0.040778018
15	0.000607717	0.000000045	0.03847882	0.0007922	0.000074053	0.020587692
20	0.000344346	0.000000012	0.02650353	0.0002952	0.000033303	0.011137033
25	0.000214045	0.000000003	0.0190409	0.0001268	0.000014016	0.006659396
30	0.000134102	0.000000001	0.01387113	0.0000544	0.000007456	0.003922285

Table S11. Lookup table (LUT) for Gel ID AW80 + IL10% (added-water 80%, theoretical $f_w = 0.8800$), showing simulated channel magnitudes at 4.5 mm and 7 mm SDS and the derived far/close ratios versus μ_s'.

μ_s' (cm^{-1})	1450 nm 4.5 mm	1450 nm 7 mm	1650 nm 4.5 mm	1650 nm 7 mm	Far/Close 1450	Far/Close 1650
0.5	0.001735498	0.00007586667	0.01354974	0.0032762	0.04371464	0.2417906
1	0.001847057	0.0000356	0.02876245	0.0068382	0.0192739	0.237734313
2	0.001675066	0.000009546	0.05853818	0.0130308	0.005698269	0.222573883
3	0.001560714	0.00000383	0.08106891	0.0156084	0.002454188	0.19255962
5	0.001514269	0.000001448	0.1027733	0.0141346	0.000956451	0.13753325
7	0.001385206	0.00000065	0.1061757	0.0095154	0.000469242	0.089614122
10	0.001111165	0.000000215	0.09611292	0.0051274	0.000193496	0.053350429
15	0.000764593	0.000000052	0.07222647	0.001922	0.000068016	0.026608441
20	0.000451835	0.000000013	0.05161793	0.0007376	0.000028772	0.014288439
25	0.000280624	0.000000004	0.03803117	0.0003288	0.000012401	0.008645321
30	0.000172042	0.000000001	0.02832466	0.000151	0.000005812	0.005331843

5. S4. DIFFUSION/SRS FORWARD MODEL USED FOR f_w CONSISTENCY-CHECK

A. Absorption mixing and diffusion parameters

In this phantom-validation configuration, absorption is assigned from the recipe-defined (nominal) water fraction rather than estimated from reflectance. In general,

$$\mu_{a,\lambda}(f_w) = \sum_i \phi_i(f_w)\,\mu_{a,i}(\lambda), \tag{S15}$$

and under the simplified water-dominant approximation used here,

$$\mu_{a,\lambda}(f_w) \approx f_w\,\mu_{a,\text{water}}(\lambda), \tag{S16}$$

with $\mu_{a,\text{water}}(\lambda)$ computed from water optical constants (e.g., Hale & Querry) using consistent length units.

$$\mu'_{t,\lambda}(f_w) = \mu_{a,\lambda}(f_w) + \hat{\mu}'_{s,\lambda}, \tag{S17}$$

$$D_\lambda(f_w) = \frac{1}{3\,\mu'_{t,\lambda}(f_w)}, \tag{S18}$$

$$\mu_{\text{eff},\lambda}(f_w) = \sqrt{3\,\mu_{a,\lambda}(f_w)\,\mu'_{t,\lambda}(f_w)}. \tag{S19}$$

B. Dipole model and objective

Define the effective source depth:

$$z_{0,\lambda}(f_w) = \frac{1}{\mu'_{t,\lambda}(f_w)}. \tag{S20}$$

Under the extrapolated boundary condition:

$$z_{b,\lambda}(f_w) = \frac{2\,D_\lambda(f_w)}{\kappa}, \tag{S21}$$

where κ depends on refractive index mismatch. Using refractive index n, define

$$a = \left(\frac{n-1}{n+1}\right)^2, \qquad b = \cos\left(\sin^{-1}\left(\frac{1}{n}\right)\right), \qquad \kappa = \frac{(1-a)(1-b^2)}{(1+a)+(1-a)b^3}. \tag{S22}$$

$$r_{1,\lambda}(\rho; f_w) = \sqrt{\rho^2 + z_{0,\lambda}^2(f_w)}, \tag{S23}$$

$$r_{2,\lambda}(\rho; f_w) = \sqrt{\rho^2 + \left(z_{0,\lambda}(f_w) + 2z_{b,\lambda}(f_w)\right)^2}. \tag{S24}$$

Modeled fluence/reflectance (dipole form):

$$U_\lambda(\rho; f_w) = \frac{C_\lambda}{4\pi D_\lambda(f_w)} \left(\frac{e^{-\mu_{\mathrm{eff},\lambda}(f_w)\, r_{1,\lambda}(\rho; f_w)}}{r_{1,\lambda}(\rho; f_w)} - \frac{e^{-\mu_{\mathrm{eff},\lambda}(f_w)\, r_{2,\lambda}(\rho; f_w)}}{r_{2,\lambda}(\rho; f_w)} \right), \tag{S25}$$

and the modeled spatial ratio:

$$r_\lambda^{\mathrm{model}}(f_w) = \frac{U_\lambda(\rho_f; f_w)}{U_\lambda(\rho_c; f_w)}. \tag{S26}$$

The objective used for the calibration-anchored water-fraction consistency check is:

$$E(f_w) = \sum_{\lambda \in \{1450, 1650\}} \left[\ln(r_\lambda^{\mathrm{corr}}) - \ln\left(r_\lambda^{\mathrm{model}}(f_w)\right) \right]^2, \tag{S27}$$

and the reported $\hat{f}_w$ is:

$$\hat{f}_w = \arg\min_{f_w \in \mathcal{F}} E(f_w). \tag{S28}$$

B.1. Model constants and refractive index

In Eq. S25, the wavelength-dependent scale factor C_λ accounts for source power, detector responsivity, and geometry-dependent coupling. Because the analysis uses *spatial ratios* at a fixed wavelength (Eq. S26), any constant multiplicative factor cancels, so only the ρ-dependence governed by μ_a and μ_s' affects $r_\lambda^{\mathrm{model}}$. Unless otherwise stated, the refractive index used for boundary terms is $n = 1.33$ (water-like), consistent with the gel's water-dominant composition.

REFERENCES

1. H. Jonasson, C. D. Anderson, and R. B. Saager, "Water and hemoglobin modulated gelatin-based phantoms to spectrally mimic inflamed tissue in the validation of biomedical techniques and the modeling of microdialysis data," *J. Biomed. Opt.* **27**(7), 074712 (2022). doi:10.1117/1.JBO.27.7.074712.
2. G. M. Hale and M. R. Querry, "Optical constants of water in the 200-nm to 200-μm wavelength region," *Appl. Opt.* **12**(3), 555–563 (1973).
3. D. Marti, R. N. Aasbjerg, P. E. Andersen, and A. K. Hansen, "MCmatlab: an open-source, user-friendly, MATLAB-integrated three-dimensional Monte Carlo light transport solver with heat diffusion and tissue damage," *J. Biomed. Opt.* **23**(12), 121622 (2018). doi:10.1117/1.JBO.23.12.121622.
4. L.-H. Wang, S. L. Jacques, and L.-Q. Zheng, "MCML—Monte Carlo modeling of light transport in multi-layered tissues," *Comput. Methods Programs Biomed.* **47**(2), 131–146 (1995). doi:10.1016/0169-2607(95)01640-F.
5. T. J. Farrell, M. S. Patterson, and B. C. Wilson, "A diffusion theory model of spatially resolved, steady-state diffuse reflectance for the noninvasive determination of tissue optical properties in vivo," *Med. Phys.* **19**(4), 879–888 (1992).
